# miR-217 and CAGE form feedback loop and regulates the response to anti-cancer drugs through EGFR and HER2

**DOI:** 10.18632/oncotarget.7185

**Published:** 2016-02-04

**Authors:** Youngmi Kim, Hyuna Kim, Deokbum Park, Minho Han, Hansoo Lee, Yun Sil Lee, Jongseon Choe, Young Myeong Kim, Dooil Jeoung

**Affiliations:** ^1^ Department of Biochemistry, College of Natural Sciences, Kangwon National University, Chunchon 200–701, Korea; ^2^ Department of Biological Sciences, College of Natural Sciences, Kangwon National University, Chunchon 200–701, Korea; ^3^ College of Pharmacy, Ewha Womans University, Seoul 03760, Korea; ^4^ Graduate School of Medicine, Kangwon National University, Chunchon 200–701, Korea

**Keywords:** anti-cancer drug-resistance, CAGE, EGFR, HER2, miR-217

## Abstract

MicroRNA array analysis revealed that miR-217 expression was decreased in anti-cancer drug-resistant Malme3M^R^ cancer cells. CAGE, a cancer/testis antigen, was predicted as a target of miR-217. Luciferase activity and ChIP assays revealed a negative feedback relationship between CAGE and miR-217. miR-217 and CAGE oppositely regulated the response to anti-cancer drugs such as taxol, gefitinib and trastuzumab, an inhibitor of HER2. miR-217 negatively regulated the tumorigenic, metastatic, angiogenic, migration and invasion potential of cancer cells. The xenograft of Malme3M^R^ cells showed an increased expression of pEGFR^Y845^. CAGE and miR-217 inhibitor regulated the expression of pEGFR^Y845^. CAGE showed interactions with EGFR and HER2 and regulated the *in vivo* sensitivity to trastuzumab. The down-regulation of EGFR or HER2 enhanced the sensitivity to anti-cancer drugs. CAGE showed direct regulation of HER2 and was necessary for the interaction between EGFR and HER2 in Malme3M^R^ cells. miR-217 inhibitor induced interactions of CAGE with EGFR and HER2 in Malme3M cells. The inhibition of EGFR by CAGE-binding GTGKT peptide enhanced the sensitivity to gefitinib and trastuzumab and prevented interactions of EGFR with CAGE and HER2. Our results show that miR-217-CAGE feedback loop serves as a target for overcoming resistance to various anti-cancer drugs, including EGFR and HER2 inhibitors.

## INTRODUCTION

miRNAs (micro RNAs) are a class of endogenous 21–23-nucleotide (in mammals) non-coding RNAs that regulate the expression of target genes either through translational inhibition or destabilization of mRNA [1–4]. miRNAs regulate tumorigenesis and cellular proliferation. miR-136 plays a tumor-suppressive role by repressing EMT and pro-metastatic traits via targeting Smad2 and Smad3 [5]. Smad2-dependent inhibition of miR-30s in podocytes is required for the activation of p53 and the induction of apoptosis by TGF-β [6]. miR-217 down-regulates the expression of a DNA damage response and repair gene network and in turn stabilizes Bcl-6 expression in germinal center (GC) B cells and also promotes mature B-cell lymphomagenesis [7]. The over expressed TGF-β1 in inflammation triggers the deregulation of the miR-217-SIRT1 pathway and then promotes the EMT process, which might be involved in the tumorigenesis of pancreatic cancer [8]. Human cytomegalo virus infection of endothelial cells induces angiogenesis by miR-217/SIRT1 and miR-217/FOXO3A axis [9]. miR-217 acts as an endogenous inhibitor of SirT1, which promotes endothelial senescence [10]. miR-217 regulates E2F3 and inhibits invasion of hepatocellular carcinoma cells [11]. miR-217 plays a tumor suppressor role in clear cell renal cell carcinoma [12]. miR-217 regulates KRAS and function as a tumor suppressor in pancreatic ductal adenocarcinoma [13]. TGF-beta activates Akt in glomerular mesangial cells by inducing miR-217, which target PTEN (phosphatase and tensin homologue), an inhibitor of Akt activation [14]. miR-217 functions as a tumor-suppressive miRNA and inhibits the osteosarcoma tumorigenesis through targeting WASF3 [15]. These reports indicate roles of miRNAs in tumorigenesis and cellular proliferation.

miRNAs regulate anti-cancer drug-resistance. For example, miR-199a suppresses tumorigenicity and multidrug resistance of ovarian cancer-initiating cells [16]. miR-27a reverses the multidrug resistance phenotype by regulating the expression of MDR1 and β-catenin [17]. The miR-200 family regulates EMT and sensitivity to paclitaxel in ovarian cancer cells [18]. Over-expression of miR-200c increases trastuzumab (Ttm) sensitivity [19]. Ph (+) leukemia cells acquire resistance to tyrosine kinase inhibitors via down-regulation of miR-217 [20]. miR-326, which is increased in anti-cancer drug-resistant cancer cells, regulates the response to anti-cancer drugs by forming a negative feedback loop with HDAC3 [21]. miR-217 acts as a tumor suppressor and regulates the resistance to cisplatin in lung cancer cells [22]. Anti-cancer drug-resistant breast cancer cells spread resistance capacity to sensitive ones by releasing exosomes and that such effect could be partly attributed to the intercellular transfer of specific miRNAs such as miR-30a [23]. miR-30a, miR-382, and miR-136 were down-regulated in bromocriptine-resistant prolactinomas in comparison with bromocriptine-sensitive prolactinomas [24]. miR-136 reverses cisplatin resistance by targeting E2F [25]. These reports indicate role of miRNAs in anti-cancer drug-resistance.

EGFR serves as a molecular target for therapy of uveal melanoma [26]. EGFR phosphorylation is associated with chemo-resistance in colorectal cancer cells [27]. The miR-200 family regulates sensitivity to EGFR therapy in bladder cancer cells [28]. CD44-EGFR interaction leads to an enhanced melanoma cell motility [29]. Because anti-cancer drug-resistance is related with the enhanced cell motility, it is plausible that EGFR may regulate the response to anti-cancer drugs. CD44 induces chemo resistance in breast cancer cells [30]. HER2 is expressed in a wide range of human melanoma cells and serves as a target for T cell mediated immunotherapy of human melanoma [31]. HER2 interaction with CD44 promotes gastric tumor progression and metastasis [32]. CD44 expression levels are higher in trastuzumab-resistant cell lines and its knockdown leads to an increased response to trastuzumab [33]. Trastuzumab- resistance is associated with the activation of EGFR signaling [34, 35]. EGFR neutralizing antibody cetuximab restores trastuzumab sensitivity of breast cancer BT474-T798M cells and xenografts, suggesting that increased EGFR ligand production was causally associated with trastuzumab resistance [36]. These reports suggest that EGFR activation is related with the resistance to EGFR inhibitor and HER2 inhibitor.

CAGE, a cancer/testis antigen, is present in the sera of gastric cancers [37], endometrial cancers [38] and patients with hematological malignancies [39]. Hypomethylation of CAGE increases the expression of CAGE [40]. CAGE displays oncogenic potential and regulates the expression of cyclins [41]. CAGE confers resistance to microtubule-targeting drugs, such as taxol and celastrol, by regulating the expression of p53 through interaction with HDAC2 [42]. miR-200b forms a negative feedback loop with CAGE and regulates the response to anti-cancer drugs [43]. miR-200c, a member of miR-200 family, shows inverse correlations with EGFR amplification [44]. DNMT1, a negative regulator of CAGE [42], reduces cancer cell proliferation and migration by inhibiting EGFR-Akt signaling [45]. This suggests the role of CAGE in EGFR signaling. miR-200s negatively regulates *in vitro* EGF/EGFR-mediated thyroid cell invasion and in EMT *in vivo* [46]. These reports suggest the role of CAGE in EGFR signaling in relation with anti-cancer drug-resistance.

In this study, we investigated the mechanism of anti-cancer drug-resistance conferred by CAGE. miR-217 and CAGE formed a negative feedback loop and oppositely regulated the response to anti-cancer drugs *in vitro* and *in vivo*. We showed that miR-217 inhibitor increased the expression of pEGFR^Y845^ and induced the interactions of CAGE with EGFR and HER2. We showed that CAGE directly regulated the expression of HER2 through its binding to the promoter sequences of HER2. Experiments employing CAGE-binding peptide showed the importance of EGFR activation in anti-cancer drug resistance conferred by CAGE. We showed that the activation of EGFR, and CAGE interactions with EGFR and HER2 were necessary for the resistance to taxol, gefitinib and trastuzumab in melanoma. Therefore miR-217-CAGE feedback loop may offer valuable target for overcoming resistance to various anti-cancer drugs such as taxol, EGFR inhibitor and HER2 inhibitor. The effect of CAGE on the response to EGFR inhibitor and HER2 inhibitor and the mechanism associated with it has not been reported.

## RESULTS

### miR-217 expression level is inversely correlated with CAGE

**Figure 1:**
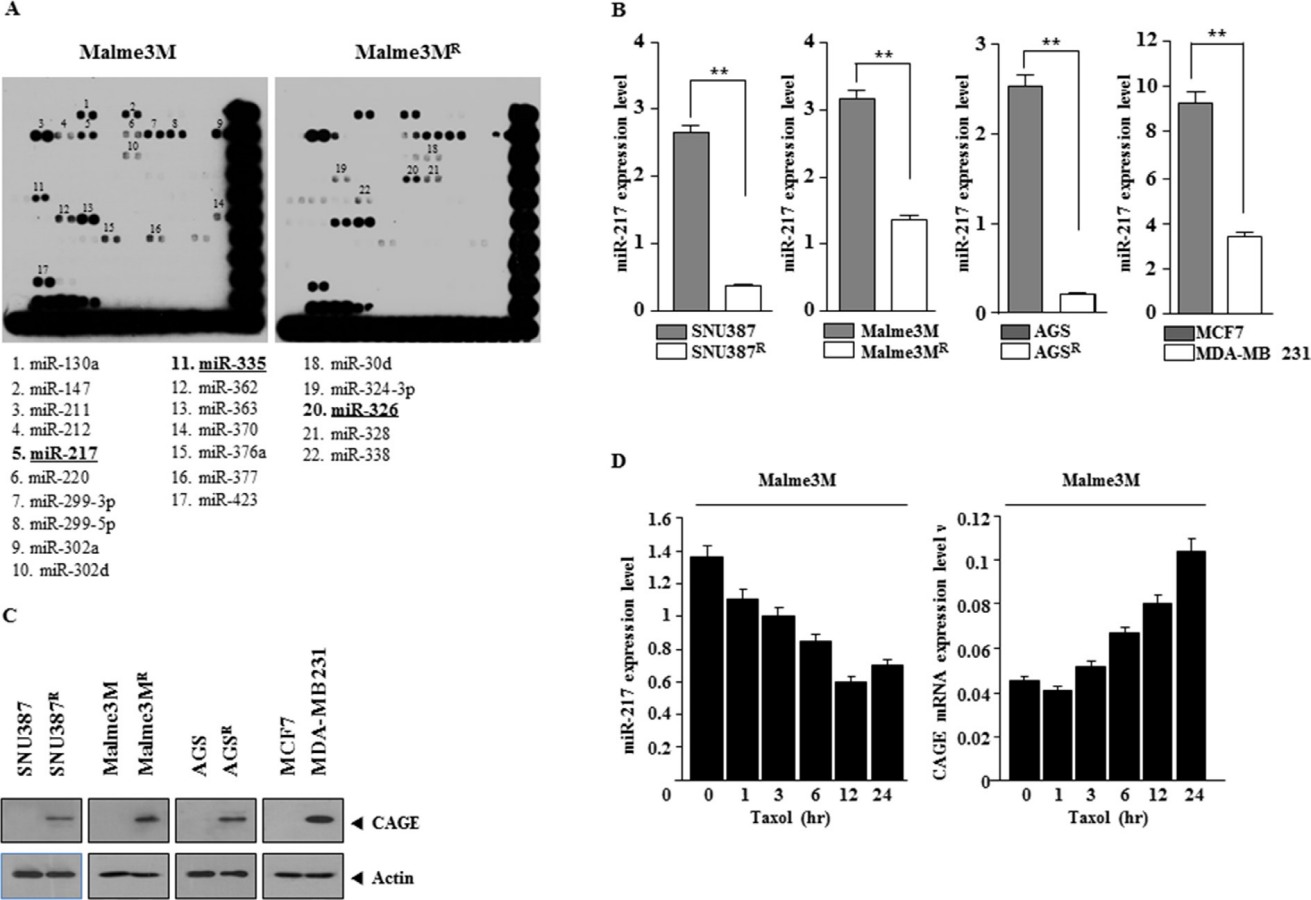
miR-217 expression level is inversely correlated with CAGE (**A**) miRNA array analysis was performed as described. Underlined miRNAs are those that show differential expression levels between Malme3M^R^ cells and Malme3M cells. (**B**) Cell lysates isolated from the indicated cancer cells were subjected to qRT-PCR analysis. **p < 0.005. (**C**) Same as (B) except that immunoblot analysis was performed. (**D**) The indicated cancer cells were treated with taxol (1 μM) for various time intervals. Cell lysate isolated at each time point were subjected to qRT-PCR analysis.

We previously reported the role of CAGE in anti-cancer drug-resistance [43]. We wanted to identify miRNAs that would regulate the expression of CAGE. miRNA array analysis showed that the expression level of miR-217 and miR-335 was lower in Malme3M^R^ cells (anti-cancer drug-resistant cancer cells) than in Malme3M cells while miR-326 showed higher expression level in Malme3M^R^ cells than in Malme3M cells (Figure 1A). miR-326 forms a feedback loop with HDAC3 and regulate the response to anti-cancer drugs [21]. Quantitative real-time PCR (qRT-PCR) showed that the expression level of miR-217 was lower in anti-cancer drug-resistant cancer cells, such as SNU387^R^, Malme3M^R^, and AGS^R^ cells (anti-cancer drug-resistant gastric cancer cells), than in SNU387, Malme3M and AGS cells (Figure 1B). The expression level of miR-217 was lower in MDA-MB231 cells, malignant breast cancer cells, than in MCF-7 cells (Figure 1B). Immunoblot blot analysis showed that the expression of CAGE, a cancer/testis antigen, was higher in Malme3M^R^, SNU387^R^, and AGS^R^ and MDA-MB-231 cells than in Malme3M, SNU387, AGS, and MCF-7 cells (Figure 1C). Anti-cancer drug, such as taxol, decreased the expression of miR-217 (Figure 1D) while increasing the expression of CAGE mRNA (Figure 1D). Taken together, these results imply that miR-217 may be associated with the response to anti-cancer drugs.

### miR-217 targets CAGE

**Figure 2:**
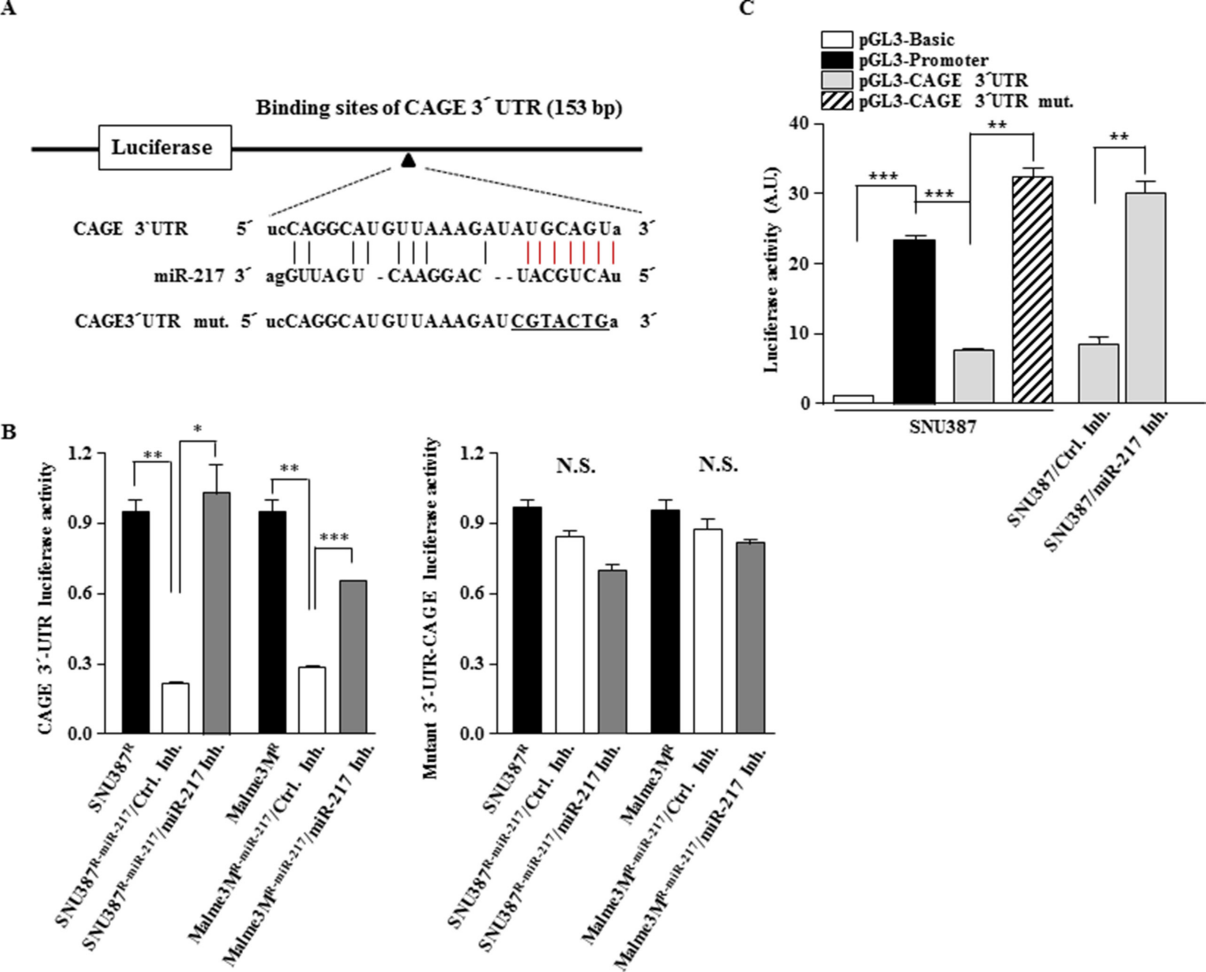
miR-217 targets CAGE (**A**) Shows the binding site of miR-217 to the 3′-UTR of CAGE. Mutant 3′-UTR-CAGE was generated by site-directed mutagenesis. Underline denotes mutated sequences. (**B**) The indicated cancer cells were transfected with the wild type 3′-UTR-CAGE or mutant 3′-UTR-CAGE (each at 1 μg) along with control inhibitor (10 nM) or miR-217 inhibitor (10 nM). At 48 h after transfection, cell lysates were prepared and subjected to luciferase activity assay. *p < 0.05; **p < 0.005; ***p < 0.005. N.S. denotes not significant. (**C**) SNU387 cells were transiently transfected with the indicated construct (each at 1 μg) along with the indicated inhibitor (10 nM). At 48 h after transfection, cell lysates were prepared and subjected to luciferase activity assay. **p < 0.005; ***p < 0.005.

Because miR-217 expression level was inversely correlated with CAGE (Figure 1B and 1C), we examined whether miR-217 would target CAGE. TargetScan analysis predicted the binding of miR-217 to the 3′-UTR of CAGE (Figure 2A). Cells that stably express miR-217 (Malme3M^R-miR-217^ and SNU387^R-miR-217^) showed lower luciferase activity associated with wild type CAGE 3′-UTR than Malme3M^R^ and SNU387^R^ cells, respectively (Figure 2B). The down-regulation of miR-217 by miR-217 inhibitor increased the luciferase activity associated with wild type 3′-UTR-CAGE, but not with mutant 3′-UTR-CAGE, in Malm3M^R-miR217^ and SNU387^R-miR-217^ cells (Figure 2B). In SNU387 cells, the luciferase activity associated with wild type 3′-UTR-CAGE was lower than the luciferase activity associated with pGL3-promoter (Figure 2C). However, SNU387 cells did not affect the luciferase activity associated with mutant 3′-UTR-CAGE (Figure 2C). miR-217 inhibitor increased the luciferase activity associated with wild type 3′-UTR-CAGE (Figure 2C). Taken together, these results suggest that miR-217 targets CAGE.

### miR-217 negatively regulates the expression of CAGE

**Figure 3:**
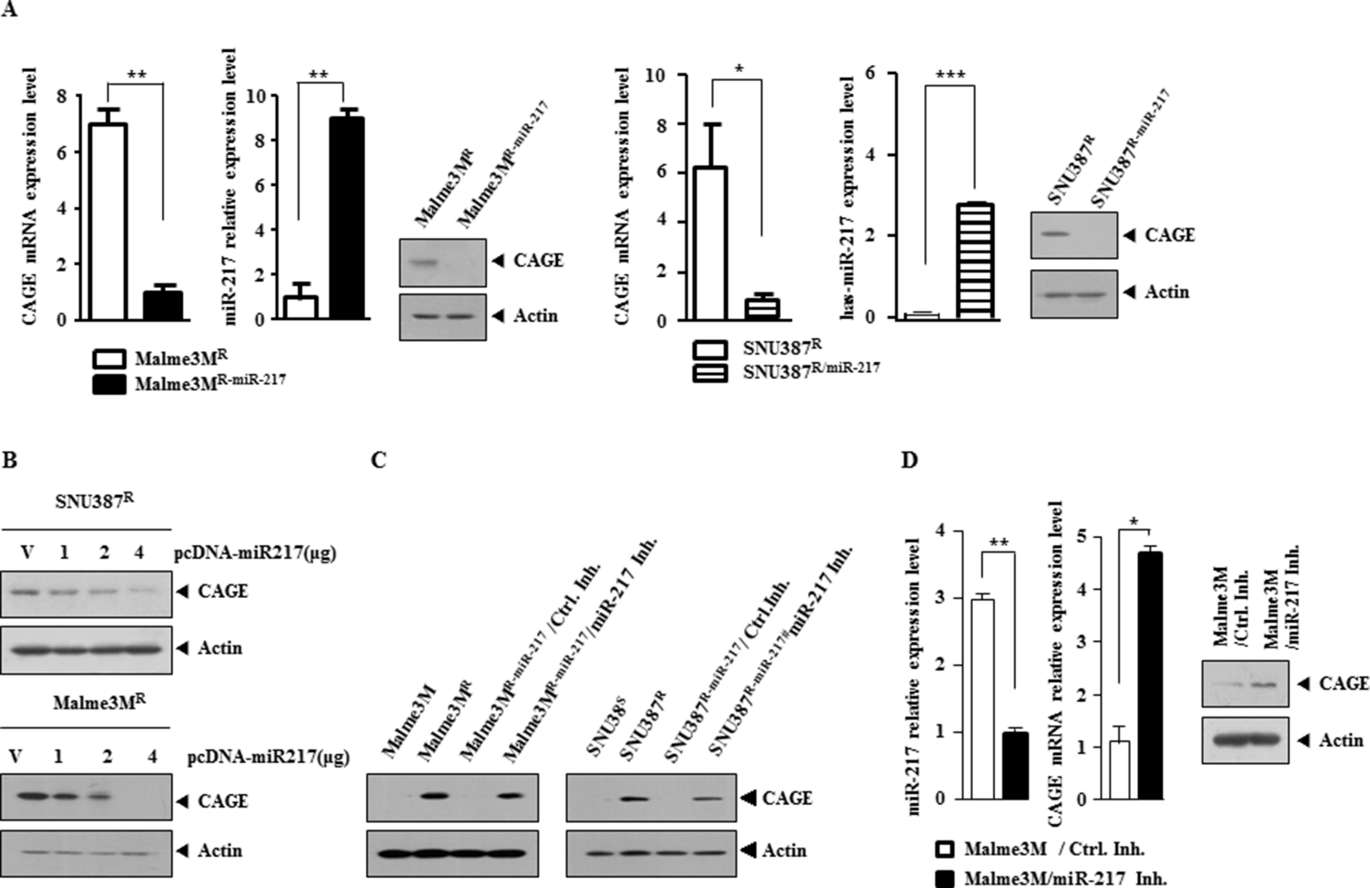
miR-217 negatively regulates the expression of CAGE (**A**) QRT-PCR analysis employing the indicated cancer cells was performed to determine the expression of CAGE and miR-217 in the indicated cancer cells. Immunoblot analysis was also performed. *p < 0.05; **p < 0.005; ***p < 0.0005. (**B**) The indicated cancer cells were transiently transfected with various concentrations of miR-217 construct. At 48 h after transfection, cell lysates were subjected to immunoblot analysis. (**C**) SNU387^R-miR-217^ or Malme3M^R-miR-217^ cells were transiently transfected with control inhibitor (10 nM) or miR-217 inhibitor (10 nM). At 48 h after transfection, cell lysates were subjected to immunoblot analysis. Cell lysates isolated from SNU387/SNU387^R^ and Malme3M/Malme3M^R^ cells were also subjected to immunoblot analysis. (**D**) Malme3M cells were transiently transfected with the control inhibitor (10 nM) or miR-217 inhibitor (10 nM). At 48 h after transfection, qRT-PCR analysis was performed to determine the expression of miR-217 and CAGE (left panel). Cell lysates were also subjected to immunoblot analysis (right panel). *p < 0.05; **p < 0.005.

We next examined the effect of miR-217 on the expression level of CAGE. Malme3M^R-miR-217^ and SNU387^R-miR-217^ cells showed lower expression level of CAGE than Malme3M^R^ and SNU387^R^ cells, respectively (Figure 3A). qRT-PCR analysis showed that Malme3M^R-miR-217^ and SNU387^R-miR-217^ cells also showed lower expression level of CAGE at the transcriptional level (Figure 3A). The over-expression of miR-217 decreased the expression of CAGE in Malme3M^R^ and SNU387^R^ cells (Figure 3B). miR-217 inhibitor increased the expression of CAGE in Malme3M^R-miR-217^ and SNU387^R-miR-217^ cells (Figure 3C). The down-regulation of miR-217 by miR-217 inhibitor in Malme3M cells increased the expression of CAGE (Figure 3D). Taken together, these results indicate that miR-217 negatively regulates the expression of CAGE.

### CAGE directly regulates the expression of miR-217

**Figure 4:**
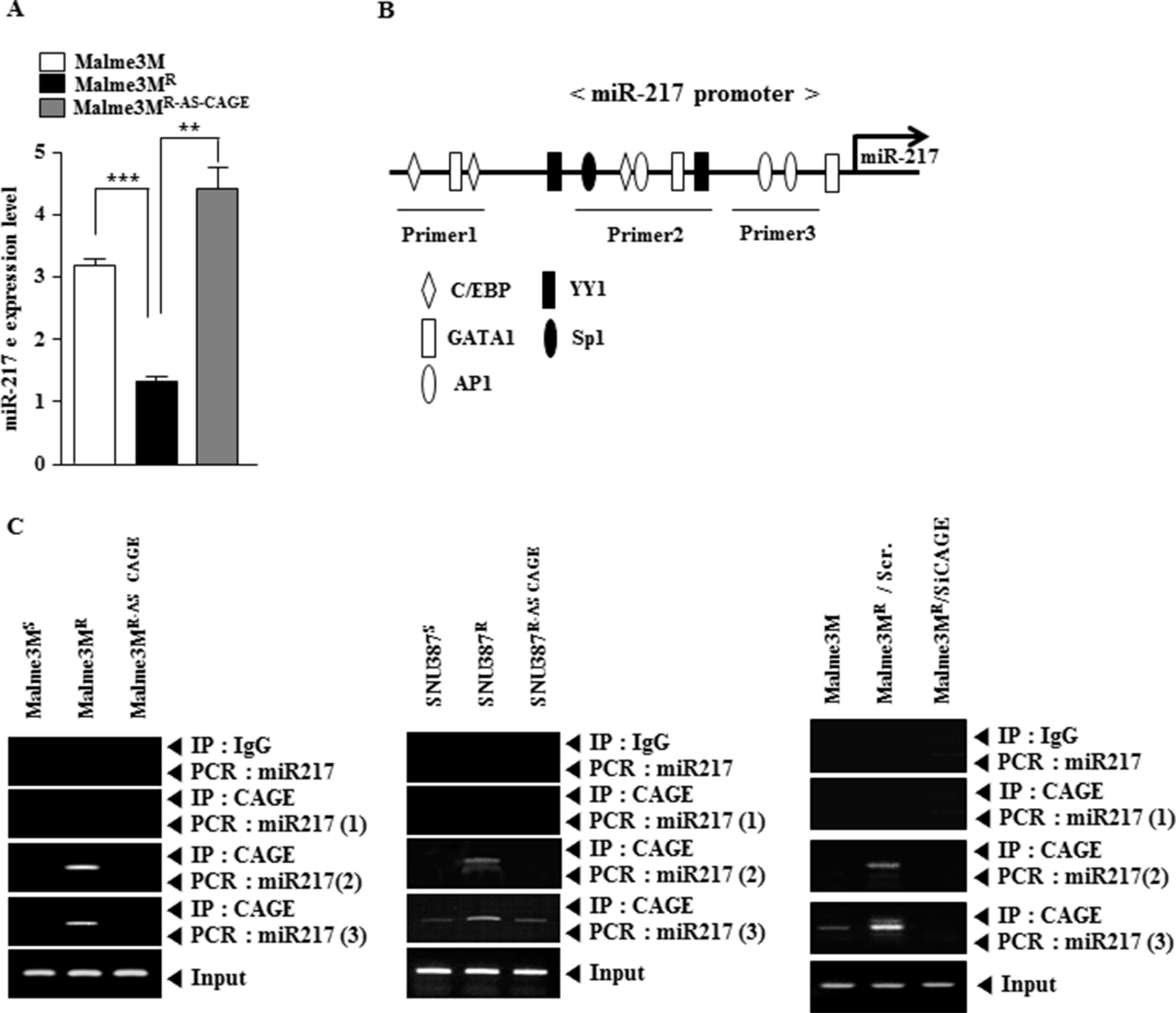
CAGE directly regulates the expression of miR-217 (**A**) Cell lysates isolated from the indicated cancer cells were subjected to qRT-PCR analysis. **p < 0.005; ***p < 0.0005. (**B**) Shows the potential binding sites of transcriptional factors in the promoter sequences of miR-217. (**C**) Cell lysates isolated from the indicated cancer cells were subjected to ChIP assays employing the indicated antibody (2 μg/ml). Malme3M^R^ cells were transiently transfected with the indicated siRNA (each at 10 nM). At 48 h after transfection, ChIP assays were also performed (right panel).

Because miR-217 expression level showed an inverse correlation with CAGE (Figure 1B and 1C), we examined the possibility of a negative feedback loop between miR-217 and CAGE. qRT-PCR analysis showed that Malme3M^R^ cells showed lower expression level of miR-217 than Malme3M cells (Figure 4A). Malme3M^R^ cells stably expressing anti-sense CAGE (Malme3M^R-AS-CAGE^) showed higher expression level of miR-217 than Malme3M^R^ cells (Figure 4B). miR-217 promoter contains binding sites for various transcription factors such as AP1, SP1, YY1, C/EBP and GATA-1 (Figure 4B). Although miR-217 promoter does not contain the potential binding site for CAGE, we examined the possibility of the direct regulation of miR-217 by CAGE. ChIP assays showed the binding of CAGE to the promoter sequences of miR-217 in Malme3M^R^ cells, but not in Malme3M^R-AS-CAGE^ cells (Figure 4C). The down-regulation of CAGE in Malme3M^R^ cells prevented CAGE from binding to the promoter sequences of miR-217 (Figure 4C). Taken together, these results suggest that miR-217 and CAGE form a negative feedback loop to regulate the response to anti-cancer drugs.

### miR-217 regulates the response to anti-cancer drugs through its effect on the expression of CAGE

**Figure 5:**
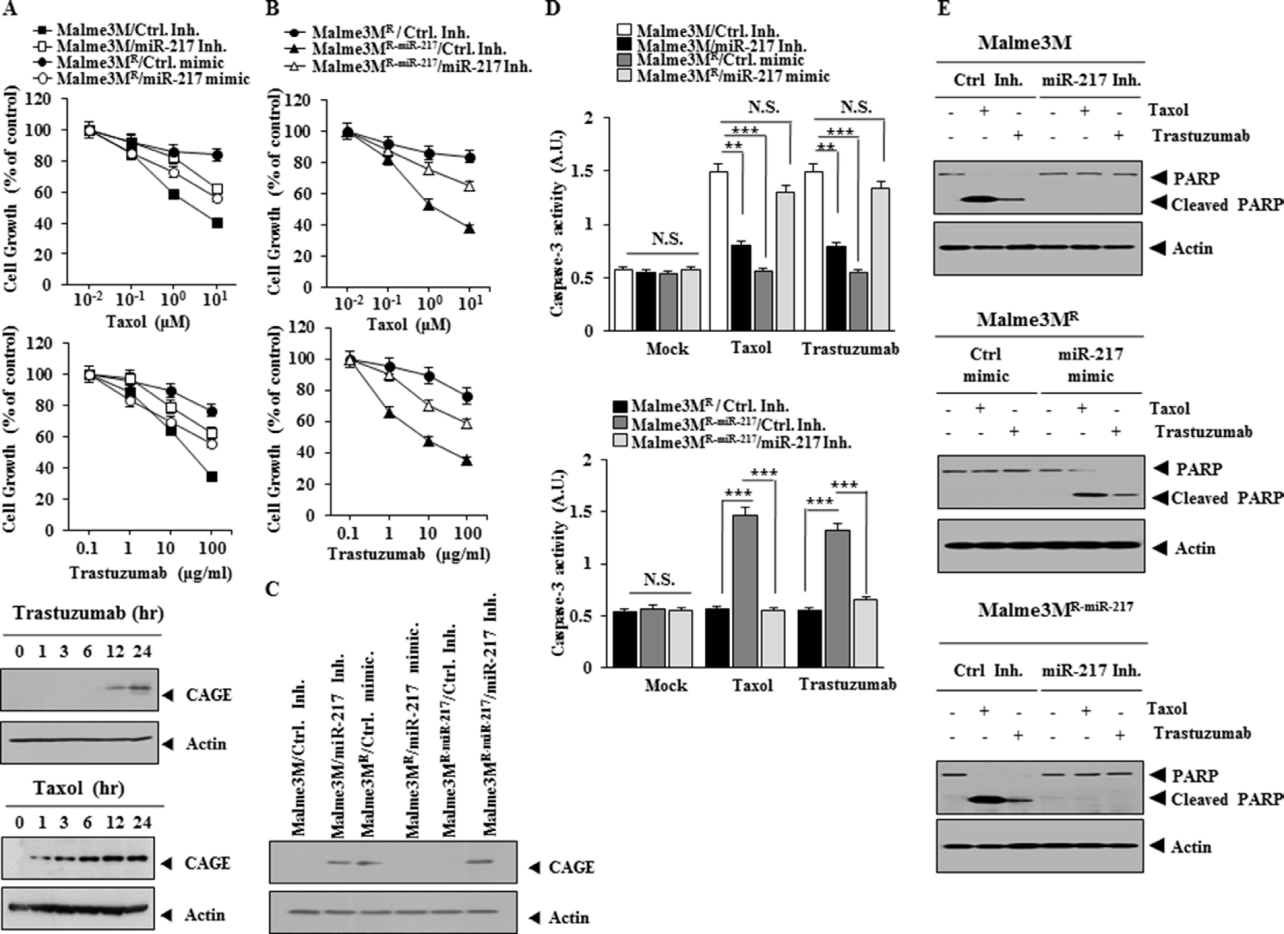
miR-217 regulates the response to anti-cancer drugs (**A**) The indicated cancer cells were transfected with the indicated inhibitor (10 nM each) or mimic (10 nM each). The next day, cells were then treated with various concentrations of taxol or trastuzumab for 24 h, followed by MTT assay. Malme3M cells were treated with trastuzumab (100 μg/ml) or taxol (1 μM) for various timer intervals. Cell lysates prepared at each time point were subjected to immunoblot analysis (lower panel). (**B**) The indicated cancer cells were transiently transfected with the indicated inhibitor (10 nM each). The next day, cells were then treated with various concentrations of taxol or trastuzumab for 24 h, followed by MTT assay. (**C**) The indicated cancer cells were transiently transfected with the indicated inhibitor (10 nM) or mimic (10 nM). At 48 h after transfection, cell lysates were isolated and subjected to immunoblot analysis. (**D**) The indicated cancer cells were transfected with the indicated inhibitor (10 nM each) or mimic (10 nM each). The next day, cells were then treated with various concentrations of taxol or trastuzumab for 24 h, followed by caspase-3 activity assays (upper panel). The indicated cancer cells were transiently transfected with the indicated inhibitor (10 nM each). The next day, cells were then treated with various concentrations of taxol or trastuzumab for 24 h, followed by caspase-3 activity assays (lower panel). **p < 0.005; ***p < 0.005. NS denotes not significant. (**E**) Malme3M cells were transiently transfected with the indicated inhibitor (10 nM each). The next day, cells were then treated with taxol (1 μM) or trastuzumab (10 μg/ml) for 24 h, followed by immunoblot analysis (upper panel). Malme3M^R^ cells were transiently transfected with the indicated mimic (10 nM each). The next day, cells were then treated with taxol (1 μM) or trastuzumab (10 μg/ml) for 24 h, followed by immunoblot analysis (middle panel). Malme3M^R-miR-217^ cells were transiently transfected with the indicated inhibitor (10 nM each). The next day, cells were then treated with taxol (1 μM) or trastuzumab (10 μg/ml) for 24 h, followed by immunoblot analysis (lower panel).

Because miR-217 regulated the expression of CAGE (Figure 3B), we examined the effect of miR-217 on the response to anti-cancer drugs. Malme3M^R^ cells, but not Malme3M cells, showed resistance to anti-cancer drugs such as taxol and trastuzumab, an inhibitor of HER2 (Figure 5A). CAGE increases the expression of cyclinD1 in A2F and AP-1-dependent manner [41]. CyclinD confers resistance to trastuzumab [47]. We hypothesized that miR-217 would regulate the response to trastuzumab through its effect on the expression of CAGE. miR-217 inhibitor conferred resistance to taxol and trastuzumab in Malme3M cells while miR-217 mimic enhanced the sensitivity to taxol and trastuzumab in Malme3M^R^ cells (Figure 5A). Taxol and trastuzumab induced the expression of CAGE in Malme3M cells (Figure 5A), suggesting that the increased expression of CAGE is associated with the resistance to taxol and trastuzumab. Malme3M^R-miR-217^ cells showed higher sensitivity to taxol and trastuzumab than Malme3M^R^ cells (Figure 5B). miR-217 inhibitor induced resistance to taxol and trastuzumab in Malme3M^R-miR-217^ cells (Figure 5B). miR-217 inhibitor increased the expression of CAGE in Malme3M cells while miR-217 mimic decreased the expression of CAGE in Malme3M^R^ cells (Figure 5C). miR-217 inhibitor restored the expression of CAGE in Malme3M^R-miR-217^ cells (Figure 5C). Malme3M cells showed higher caspase-3 activity in response to taxol and trastuzumab than Malme3M^R^ cells (Figure 5D). miR-217 inhibitor exerted a negative effect on the increased caspase-3 activity in Malme3M cells in response to taxol and trastuzumab while miR-217 mimic enhanced caspase-3 activity in Malme3M^R^ cells in response to taxol and trastuzumab (Figure 5D). miR-217 inhibitor prevented the cleavage of PARP in Malme3M cells and Malme3M^R-miR-217^ cells in response to taxol and trastuzumab while miR-217 mimic induced the cleavage of PARP in Malme3M^R^ cells in response to taxol and trastuzumab (Figure 5E). Taken together, these results suggest that miR-217 regulates the response to anti-cancer drugs in a manner associated with its effect on the expression of CAGE.

### miR-217 and CAGE cross regulate each other and exert opposite regulations on the response to anti-cancer drugs

**Figure 6:**
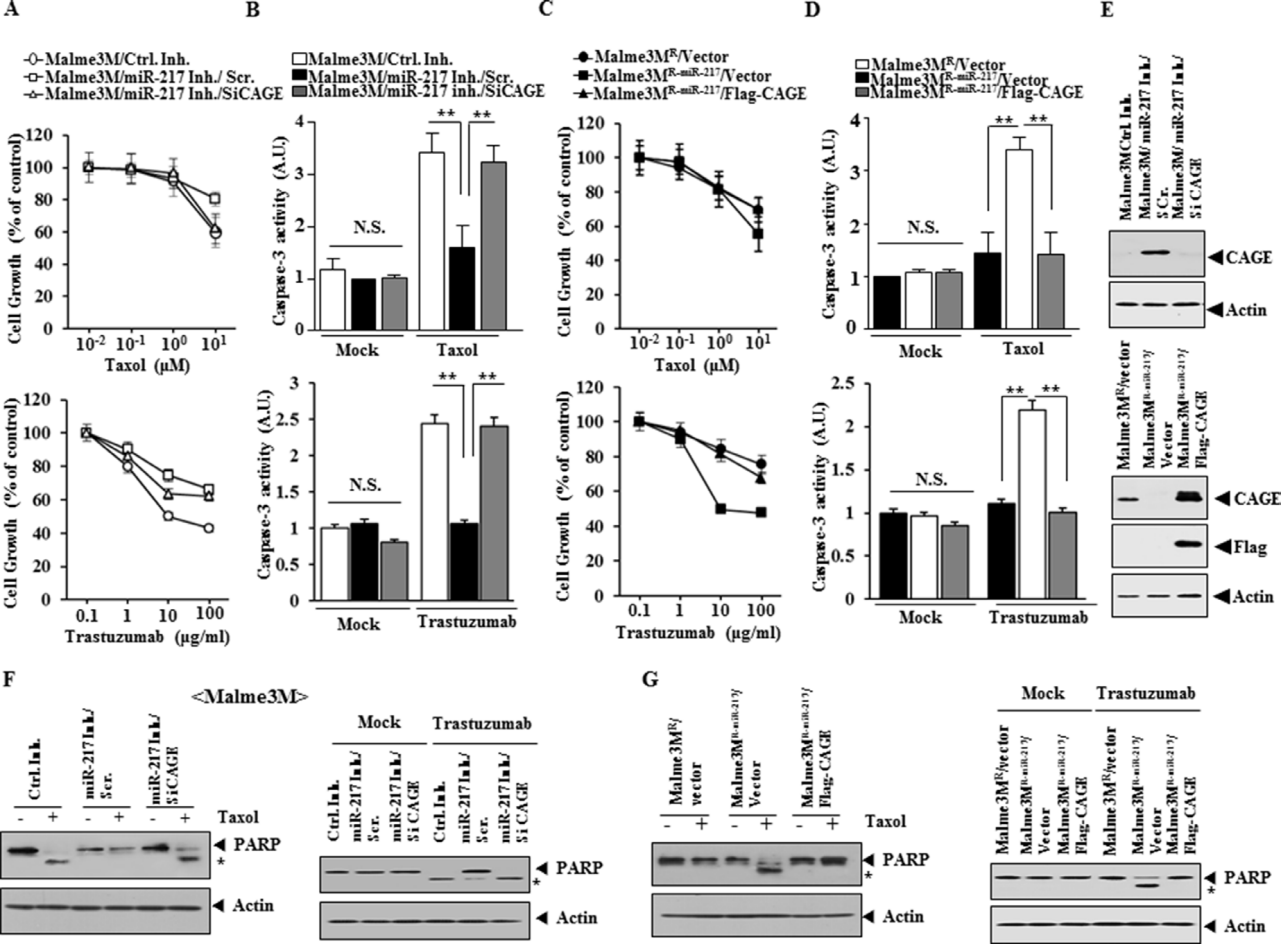
miR-217 and CAGE cross regulate each other and exert opposite regulations on the response to anti-cancer drugs (**A**) Malme3M cells were transiently transfected with the indicated inhibitor (10 nM) along with the indicated siRNA (10 nM). The next day, cells were then treated with various concentrations of taxol or trastuzumab for 24 h, followed by MTT assays. (**B**) Same as (A) except that cells were treated with taxol (1 μM) or trastuzumab (100 μg/ml), followed by caspase-3 activity assays. **p < 0.005. (**C**) The indicated cancer cells were transiently transfected with control vector (1 μg) or Flag-CAGE (1 μg). The next day, cells were then treated with various concentrations of taxol or trastuzumab, followed by MTT assays. (**D**) Same as (C) except that cells were treated with taxol (1 μM) or trastuzumab (100 μg/ml), followed by caspase-3 activity assays. **p < 0.005. (**E**) The indicated cancer cells were transiently transfected with the indicated inhibitor (10 nM), siRNA (10 nM) or vector (1 μg). At 48 h after transfection, cell lysates were prepared and subjected to immunoblot analysis. (**F**) Same as (B) except that immunoblot analysis was performed. (**G**) Same as D except that immunoblot analysis was performed.

miR-217 inhibitor conferred resistance to taxol and trastuzumab in Malme3M cells (Figure 6A). The down-regulation of CAGE by siRNA prevented miR-217 inhibitor from conferring resistance to taxol and trastuzumab in Malme3M cells (Figure 6A). miR-217 inhibitor prevented taxol and trastuzumab from increasing caspase-3 activity in Malme3M cells in CAGE-dependent manner (Figure 6B). Malme3M^R-miR-217^ cells showed higher sensitivity to taxol and trastuzumab than Malme3M^R^ cells (Figure 6C). CAGE conferred resistance to taxol and trastuzumab in Malme3M^R-miR-217^ cells (Figure 6C). Malme3M^R-miR-217^ cells, but not Malme3M^R^ cells, showed increased caspase-3 activity in response to taxol and trastuzumab (Figure 6D). CAGE prevented taxol and trastuzumab from increasing caspase-3 activity in Malme3M^R-miR-217^ cells (Figure 6D). miR-217 specifically regulated the expression of CAGE (Figure 6E). miR-217 inhibitor prevented taxol and trastuzumab from cleaving PARP in Malme3M cells in CAGE-dependent manner (Figure 6F). CAGE prevented taxol and trastuzumab from increasing caspase-3 activity in Malme3M^R-miR-217^ cells (Figure 6G). Taken together, these results suggest that miR-217 and CAGE form a negative feedback loop to regulate the response to anti-cancer drugs.

### Malme3M^R-miR-217^ cells show lower tumorigenic potential and angiogenic potential than Malme3M^R^ cells

**Figure 7:**
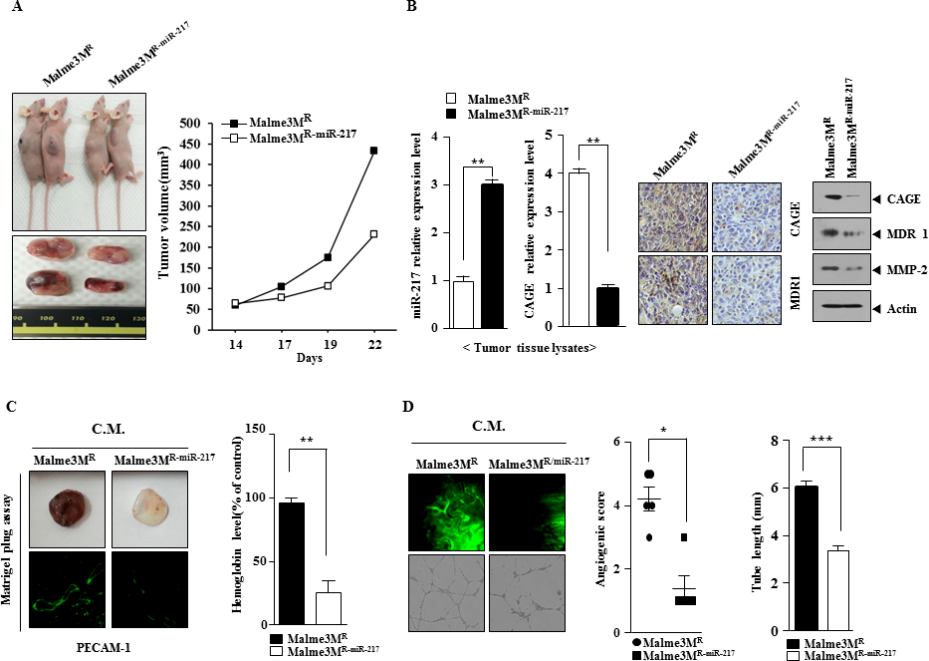
Malme3M^R-miR-217^ cells show lower tumorigenic and angiogenic potential than Malme3M^R^ cells (**A**) The tumorigenic potential of Malme3M^R^ and Malme3M^R-miR-217^ cells was determined as described. (**B**) Tumor tissue lysate were isolated and subjected to qRT-PCR analysis to determine the expression level of CAGE and miR-217. Tumor tissue lysates were subjected to immunoblot analysis. Immunohistochemistry staining of tumor tissue employing anti-CAGE antibody was performed as described. Tumor tissue sections were counterstained with hematoxylin. **p < 0.005. (**C**) The conditioned medium obtained from the indicated cancer cells were mixed with matrigel, followed by matrigel plug assays. Immunofluorescence staining of the matrigel was performed to determine the expression of PECAM-1, a marker of angiogenesis. **p < 0.005. C.M. denotes conditioned medium. (**D**) The conditioned medium obtained from the indicated cancer cells were subjected to intravital microscopy (upper panel) and endothelial cell tube formation assay (lower panel). *p < 0.05; ***p < 0.0005.

Because miR-217 regulated the response to anti-cancer drugs, we examined the effect of miR-217 on the tumorigenic and angiogenic potential of cancer cells. The *in vivo* xenograft of Mame3M^R^ cells showed higher tumorigenic potential than the xenograft of Malme3M^R-miR-217^ cells (Figure 7A). Malme3M^R-miR-217^ cells showed lower expression level of CAGE than Malme3M^R^ cells in qRT-PCR analysis (Figure 7B). Immunoblot analysis of tumor tissue lysates showed that Malme3M^R-miR-217^ cells expressed lower level of CAGE, MDR1 and MMP-2 than Malme3M^R^ cells (Figure 7B). Immunohistochemistry staining of tumor tissues showed that Malme3M^R-miR-217^ cells expressed lower level of CAGE than Malme3M^R^ cells (Figure 7B). Matrigel plug assay employing the conditioned medium showed that Malme3M^R-miR217^ cells displayed lower angiogenic potential than Malme3M^R^ cells (Figure 7C). Intravital microscopy and human endothelial cell tube formation assays employing the conditioned medium also showed that Malme3M^R-miR217^ cells displayed lower angiogenic potential than Malme3M^R^ cells (Figure 7D). Taken together, these results suggest that miR-217 negatively regulate the tumorigenic and angiogenic potential of cancer cells in relation with its effect on the response to anti-cancer drugs.

### miR-217 negatively regulates the metastatic potential of Malme3M^R^ cells

We next examined the effect of miR-217 on the metastatic potential of cancer cells. Malme3M^R^ cells showed higher metastatic potential than Malme3M^R-miR-217^ cells (Supplementary Figure S1A). miR-217 mimic decreased the metastatic potential of Malme3M^R^ cells (Supplementary Figure S1A). Immunohistochemistry staining of lung tumor tissue showed that miR-217 mimic decreased the expression of CAGE in the xenograft of Malme3M^R^ cells (Supplementary Figure S1A). qRT-PCR analysis showed that miR-217 mimic decreased the expression of CAGE in Malme3M^R^ cells (Supplementary Figure S1B). Lung tumor tissue from Malme3M^R-miR-217^ cells showed lower expression level of CAGE and MDR1 than lung tumor tissue from Malme3M^R^ cells (Supplementary Figure S1B). Immunoblot showed that miR-217 mimic decreased the expression of CAGE and MDR1 in the xenograft of Malme3M^R^ cells (Supplementary Figure S1 B). Malme3M^R^ cells showed higher metastatic potential than Malme3M^R-miR-217^ cells (Supplementary Figure S1C). miR-217 inhibitor enhanced the metastatic potential of Malme3M^R-miR-217^ cells (Supplementary Figure S1C). qRT-PCR analysis showed that miR-217 inhibitor restored the expression of CAGE in the xenograft of Malme3M^R-miR-217^ cells (Supplementary Figure S1D). Immunoblot analysis of tumor tissue lysates showed that miR-217 inhibitor restored the expression of CAGE and MDR1 in Malme3M^R-miR-217^ cells (Supplementary Figure S1D). Taken together, these results suggest that miR-217 negatively regulates the metastatic potential of cancer cells in a manner associated with its effect on the expression of CAGE.

### miR-217 inhibitor enhances the tumorigenic, metastatic and angiogenic potential of Malme3M cells

**Figure 8:**
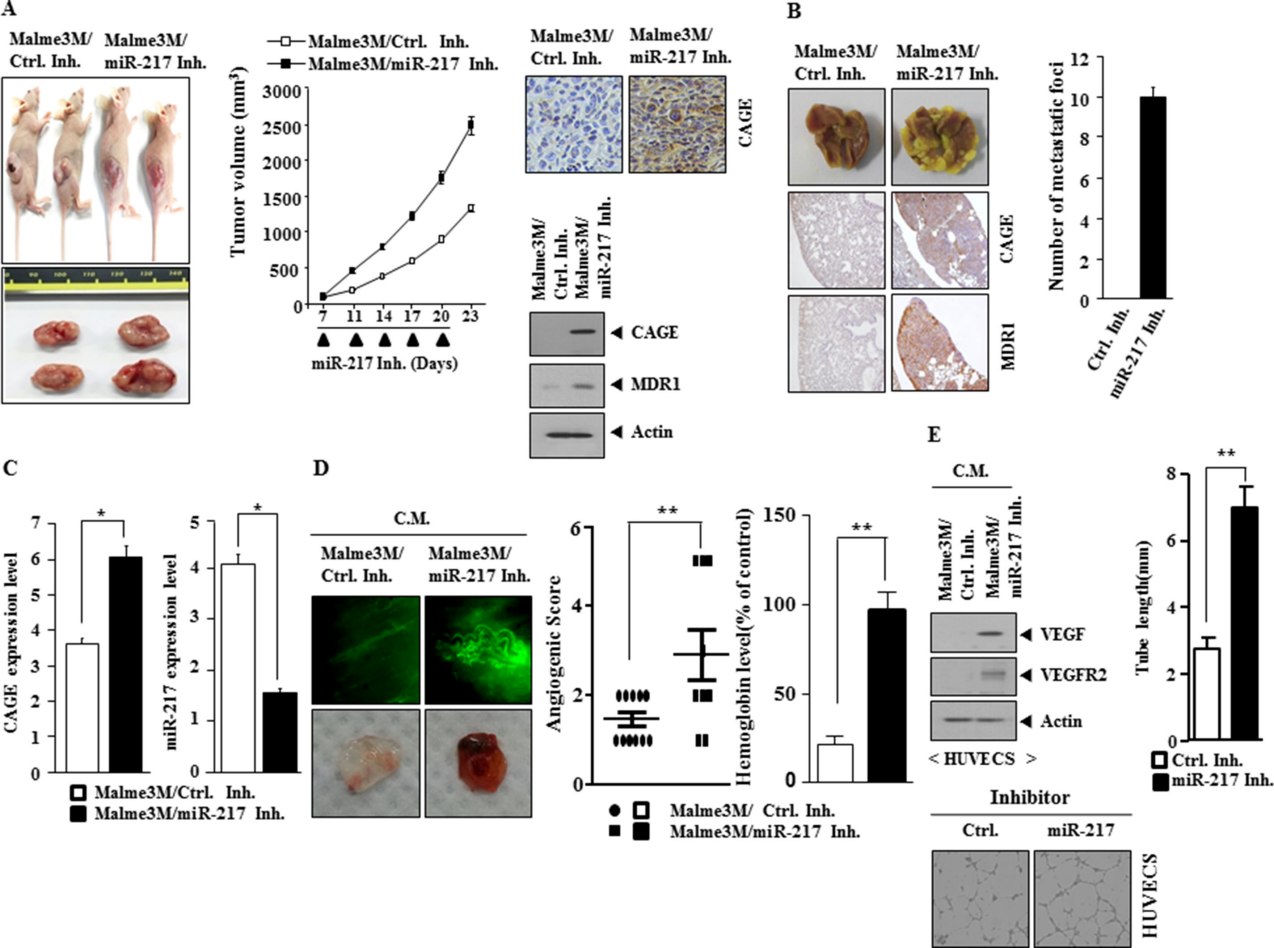
miR-217 inhibitor increases the tumorigenic potential, metastatic potential and angiogenic potential of Malme3M cells (**A**) Malme3M cells (10^6^) were injected into the dorsal flanks of athymic nude mice. Control inhibitor (50 μM/kg) or miR-217 inhibitor (50 μM/kg) was intravenously injected five times in a total of 23 days. The extent of tumorigenic potential was determined as described. Immunohistochemistry staining and immunoblot tumor tissue were performed to determine the effect of miR-217 inhibitor on the expression of CAGE. (**B**) Control inhibitor (50 μM/kg) or miR-217 inhibitor (50 μM/kg) was intravenously injected five times over a total of 4 weeks. The extent of lung metastases was determined by staining with Bouin's solution. The immunohistochemistry staining of lung tumor tissue employing the indicated antibody (2 μg/ml) was performed. (**C**) Lysates isolated from lung tumor tissue were subjected to qRT-PCR analysis to determine the expression of CAGE and miR-217. *p < 0.05. (**D**) The conditioned medium of Malme3M cells transfected with control inhibitor (10 nM) or miR-217 inhibitor (10 nM) was subjected to intravital microscopy (upper panel) and matrigel plug assay (lower panel). **p < 0.005. (**E**) The conditioned medium of Malme3M cells transfected with control inhibitor (10 nM) or miR-217 inhibitor (10 nM) was added to HUVECs for 1 h, followed by immunoblot analysis. The conditioned medium was also subjected to endothelial cell tube formation assays employing HUVECs. **p < 0.005.

Because miR-217 mimic decreased the metastatic potential of Malme3M^R^ cells (Supplementary Figure S1A), we examined whether miR-217 inhibitor would enhance the tumorigenic, metastatic and angiogenic potential of Malme3M cells. miR-217 inhibitor enhanced the tumorigenic potential of Malme3M cells (Figure 8A). Immunohistochemistry staining and immunoblot showed that miR-217 inhibitor increased the expression of CAGE (Figure 8A). miR-217 inhibitor enhanced the metastatic potential of Malme3M cells (Figure 8B). Immunohistochemistry staining of tumor tissue showed that miR-217 inhibitor increased the expression of CAGE and MDR1 (Figure 8B). qRT-PCR analysis of tumor tissue lystes showed that miR-217 inhibitor increased the expression of CAGE (Figure 8C). Matrigel plug and intravital microscopy analysis employing the conditioned medium showed that miR-217 inhibitor enhanced the angiogenic potential of Malme3M cells (Figure 8D). The conditioned medium of Malme3M cells transfected with miR-217 inhibitor, when added to HUVECs, increased the expression of VEGF and VEGFR2 (Figure 8E), and enhanced the endothelial cell tube formation (Figure 8E). Taken together, these results suggest that miR-217 inhibitor enhances the tumorigenic, metastatic and angiogenic potential in a manner associated with its effect on the expression of CAGE.

### miR-217-CAGE negative feedback loop regulates the invasion and migration potential of cancer cells

miR-217 inhibitor enhanced the invasion and migration potential of Malme3M cells in CAGE-dependent manner (Supplementary Figure S2A). miR-217 inhibitor specifically regulated the expression of CAGE in Malme3M cells (Supplementary Figure S2B). Malme3M^R-miR-217^ cells showed lower invasion and migration potential than Malme3M^R^ cells (Supplementary Figure S2C). CAGE enhanced the invasion and migration potential of Malme3M^R-miR-217^ cells (Supplementary Figure S2C). Malme3M^R-miR-217^ cells showed lower expression level of CAGE than Malme3M^R^ cells (Supplementary Figure S2D). Taken together, these results suggest that miR-217 and CAGE form a negative feedback loop to regulate the invasion and migration potential of cancer cells.

### CAGE interacts with EGFR and is necessary for the increased phosphorylation of EGFR in Malme3M^R^ cells

**Figure 9:**
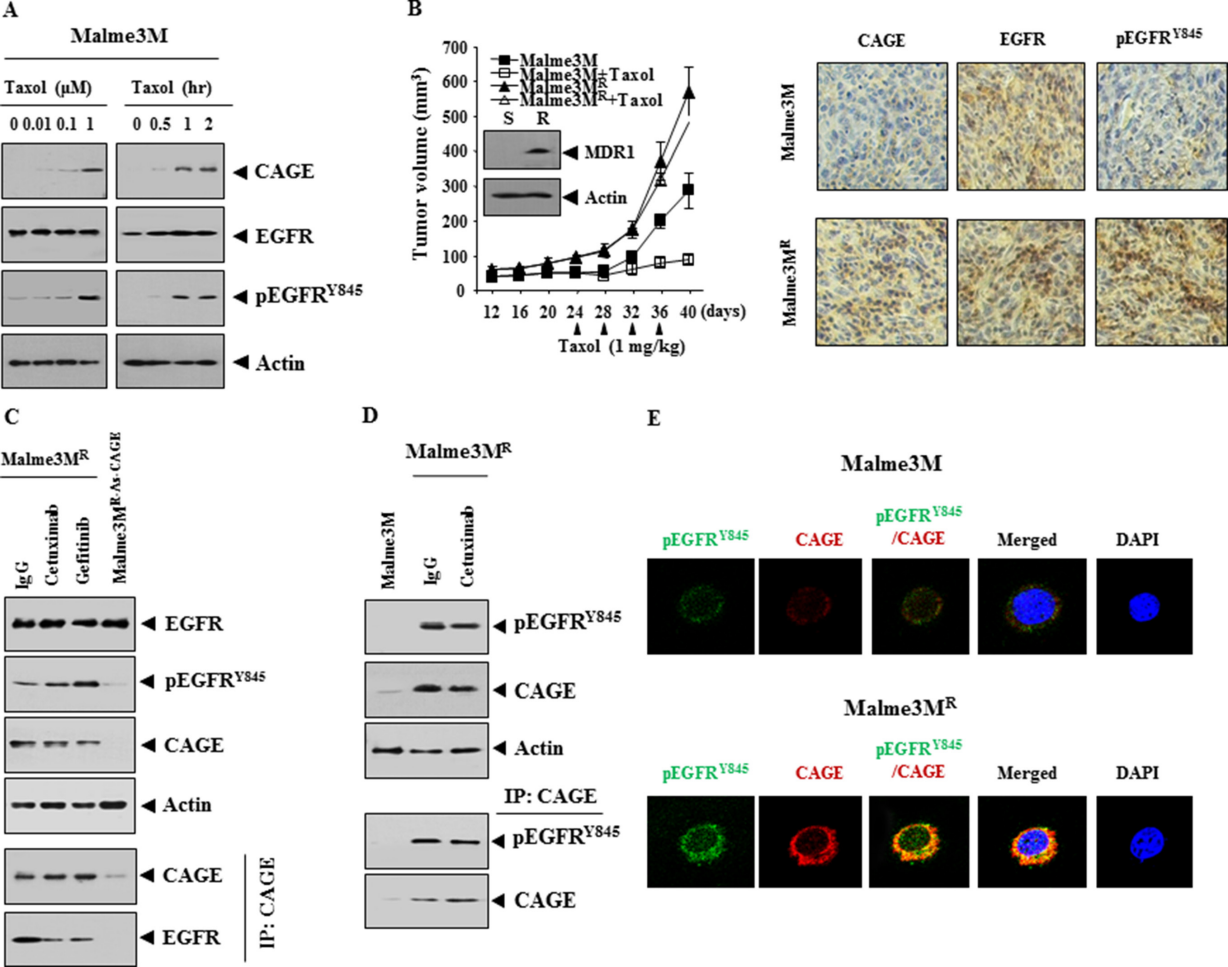
CAGE interacts with EGFR and is necessary for the increased phosphorylation of EGFR in Malme3M^R^ cells (**A**) Malme3M cells were treated with taxol (1 μM) for various time intervals or cells were treated with various concentrations of taxol for 24 h. Cell lysates were subjected to immunoblot analysis. (**B**) Malme3M (1 × 10^6^) or Malme3M^R^ cells (1 × 10^6^) were injected into the dorsal flank area of athymic nude mouse. Taxol (1 mg/kg) was injected into each nude mouse after the tumor reached a certain size (∼50 mm^3^). Tumor volume was measured on the same day as injection of taxol. Tumor-bearing mice were assessed for weight loss. Five mice were used for the injection of each cell line. Each value represents an average obtained from five mice of each group. Data are expressed as mean ± SD. Immunoblot of tumor lysates was performed. Immunohistochemistry staining (right panel) of tumor tissue derived from Malme3M or Malme3M^R^ cells was performed as described. Paraffin sections (4–6 μm thickness) of the tumor tissues were stained with the indicated antibodies. Immunohistochemistry staining employing secondary antibody alone served as a negative control. Representative images from five animals from each experimental group are shown (magnification, 400X; Olympus). H & E staining was performed to check structural integrity. All animal experiments were approved by Institutional review Board for animal studies of Kangwon National University. (**C**) Malme3M^R^ cells were treated with IgG (10 μg/ml), cetuximab (10 μg/ml) or gefitinib (1 μM) for 24 h. Cell lysates prepared were subjected to immunoblot and immunoprecipitation analysis. Cell lysates isolated from Malme3M^R-As-CAGE^ cells that stably express anti-sense CAGE were also subjected to immunoblot and immunoprecipitation analysis. (**D**) Malme3M^R^ cells were treated with IgG (10 μg/ml) or cetuximab (10 μg/ml) for 24 h. Cell lysates were immunoprecipitated with the indicated antibody (2 μg/ml), followed by immunoblot analysis (lower panel). Cell lysates were also subjected to immunoblot analysis (upper panel). (**E**) Immunofluorescence staining employing the indicated antibody (2 μg/ml) was performed as described.

Reportedly, taxol increases the phosphorylation of EGFR [27], suggesting the role of EGFR in the response to anti-cancer drugs. Taxol increased the expression of CAGE and pEGFR^Y845^ in Malme3M cells (Figure 9A). Celastrol, a microtubule-targeting anti-cancer drug, increased the expression of Malme3M cells (data not shown). The xenograft of Malme3M^R^ cells showed an increased expression of MDR1 and resistance to taxol (Figure 9B). Immunohistochemistry staining of xenograft of Malme3M^R^ cells showed the increased expression of CAGE and pEGFR^Y854^ (Figure 9B). EGFR inhibitors, such as cetuximab and gefitinib, did not affect the expression of pEGFR^Y854^ or CAGE while stable over-expression of anti-sense CAGE decreased the expression of pEGFR^Y854^, but not the expression of EGFR, in Malme3M^R^ cells (Figure 9C). CAGE showed an interaction with EGFR (Figure 9C) and pEGFR^Y845^ in Malme3M^R^ cells (Figure 9D). Immunofluorescence staining showed a co-localization of CAGE with pEGFR^Y845^ (Figure 9E). Taken together, these results suggest the role of EGFR in anti-cancer drug-resistance conferred by CAGE.

### CAGE confers resistance to EGFR inhibitors and increases the expression of pEGFR^Y845^

**Figure 10:**
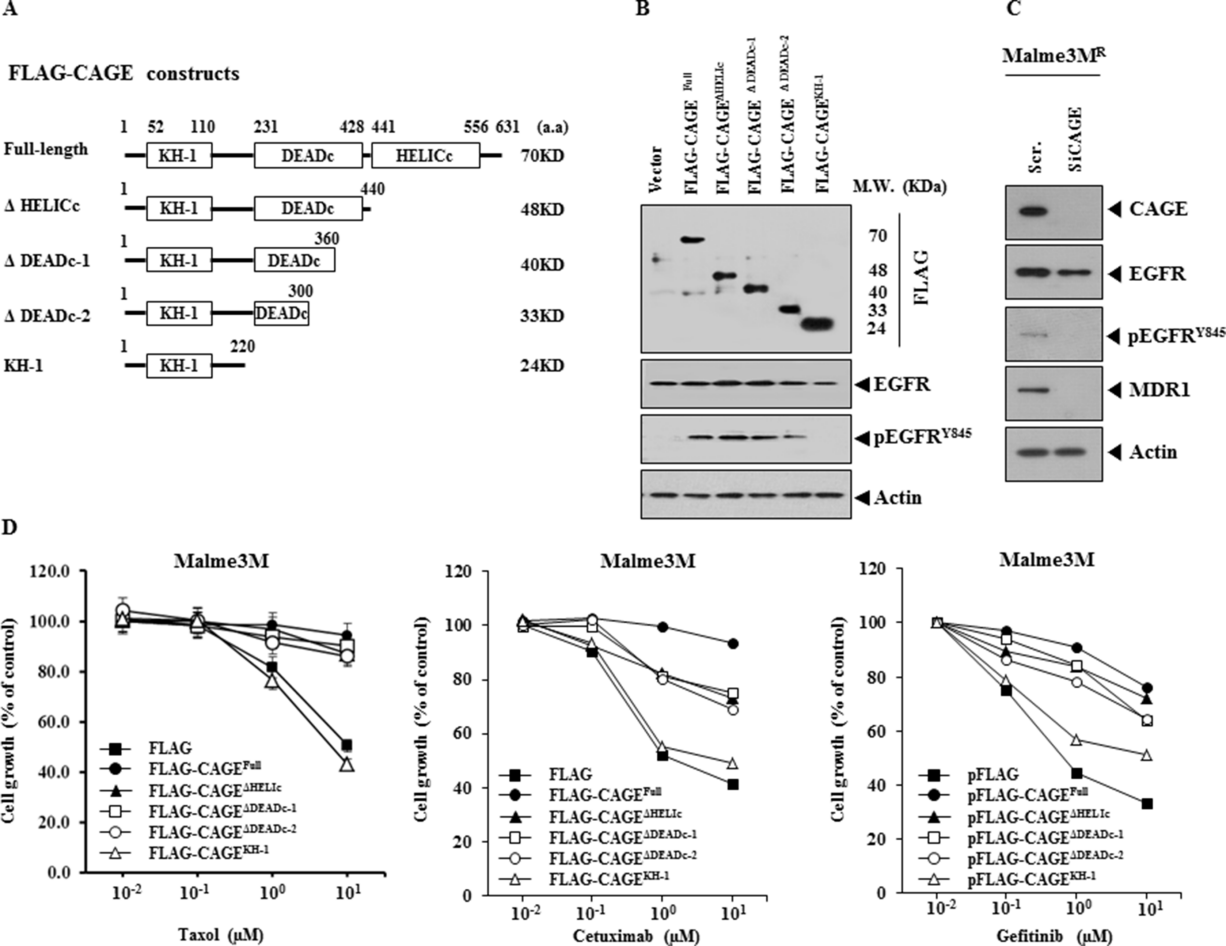
CAGE confers resistance to EGFR inhibitors (**A**) Shows CAGE deletion constructs. (**B**) Each construct (1 μg) was transiently transfected into Malme3M cells. At 48 h after transfection, cell lysates were immunoprecipitated with the indicated antibody (2 μg/ml), followed by immunoblot analysis. Cell lysates were also subjected to immunoblot analysis, (**C**) Malme3M^R^ cells were transiently transfected with the indicated siRNA (each at 10 nM). At 48 h after transfection, cell lysates were subjected to immunoblot analysis. (**D**) Each construct (1 μg) was transiently transfected into Malme3M cells. At 24 h after transfection, cells were then treated with the indicated anti-cancer drugs for 24 h, followed by MTT assays.

The down-regulation of CAGE led to the decreased expression of pEGFR^Y845^ in Malme3M^R^ cells (Figure 9C). This led us to hypothesize that CAGE would confer resistance to EGFR inhibitors. We determined the domain necessary for conferring resistance to anti-cancer drugs. For this, we made a series of CAGE deletion constructs (Figure 10A). These constructs increased the expression of pEGFR^Y845^, but not the expression of EGFR, in Malme3M cells (Figure 10B). However, CAGE deletion construct that completely lacks DEAD box domain (KH-1) did not induce the expression of pEGFR^Y845^ in Malme3M cells (Figure 10B). The down-regulation of CAGE decreased the expression of pEGFR^Y845^ and MDR1 in Malme3M^R^ cells (Figure 10C). With the exception of KH-1construct, CAGE constructs conferred resistance to taxol and EGFR inhibitors such as cetuximab and gefitinib (Figure 10D). CAGE constructs also conferred resistance to celastrol (data not shown). Taken together, these results suggest that CAGE regulates the response to EGFR inhibitors through its effect on the expression of pEGFR^Y845^.

### The down-regulation of EGFR leads to the enhanced the sensitivity to anti-cancer drugs

**Figure 11:**
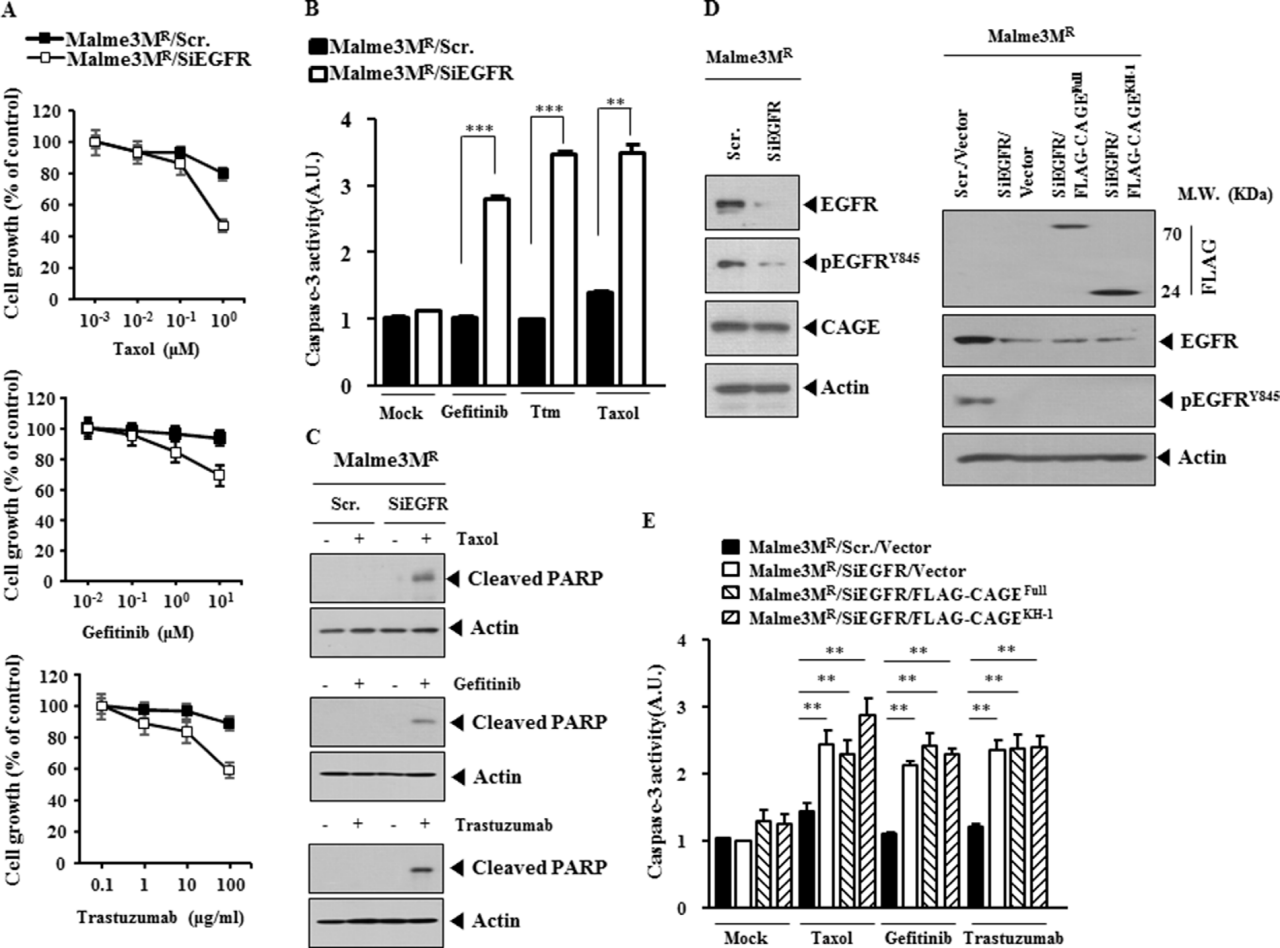
The down-regulation of EGFR enhances sensitivity to anti-cancer drugs (**A**) Malme3M^R^ cells were transiently transfected with the indicated siRNA (each at 10 nM). At 24 h after transfection, cells were then with the indicated anti-cancer drugs for 24 h, followed by MTT assays. (**B**) Malme3M^R^ cells were transiently transfected with the indicated siRNA (10 nM). At 24 h after transfection, cells were then with taxol (1 μM), gefitinib (1 μM) or trastuzumab (10 μg/ml) for 24 h, followed by caspase-3 activity assays. Ttm denotes trastuzumab. **p < 0.005; ***p < 0.0005. (**C**) Same as (B) except that immunoblot analysis was performed. (**D**) Malme3M^R^ cells were transiently transfected with the indicated siRNA (each at 10 nM). At 48 h after transfection, cell lysates were subjected to immunoblot analysis (left panel). Malme3M^R^ cells were transiently transfected with the indicated siRNA (10 nM) along with the indicated construct (1 μg). At 48 h after transfection, immunoblot analysis was performed (right panel). (**E**) Malme3M^R^ cells were transiently transfected with the indicated siRNA (10 nM) along with the indicated construct (1 μg). At 24 h after transfection, cells were then treated with taxol (1 μM), gefitinib (1 μM) or trastuzumab (10 μg/ml) for 24 h, followed by caspase-3 activity assays. **p < 0.005.

We next examined the effect of EGFR on the response to anti-cancer drugs. The down-regulation of EGFR enhanced the sensitivity to taxol, gefitinib and trastuzumab (Figure 11A), increased caspase-3 activity in response to these anti-cancer drugs in Malme3M^R^ cells (Figure 11B), but not the expression of CAGE or HER2 (Figure 11B), and induced cleavage of PARP in response to taxol, gefitinib and trastuzumab in Malme3M^R^ cells (Figure 11C). These results suggest that EGFR mediates anti-cancer drug-resistance conferred by CAGE. The down-regulation of EGFR decreased the expression of pEGFR^Y845^, but not the expression of CAGE (Figure 11D). Overexpression of CAGE in Malme3M^R^ cells transfected with siEGFR did not restore the expression of EGFR or pEGFR^Y845^ (Figure 11D). The down-regulation of EGFR increased caspase-3 activity in response to taxol, gefitinib and trastuzumab in Malme3M^R^ cells (Figure 11E). Full-length CAGE did not prevent caspase-3 activity from increasing, in response to anti-cancer drugs, in Malme3M^R^ cells transfected with siEGFR (Figure 11E). Taken together, these results suggest that EGFR mediates anti-cancer drug-resistance conferred by CAGE.

### CAGE interacts with HER2, directly regulates the expression of HER2 and confers resistance to trastuzumab

**Figure 12:**
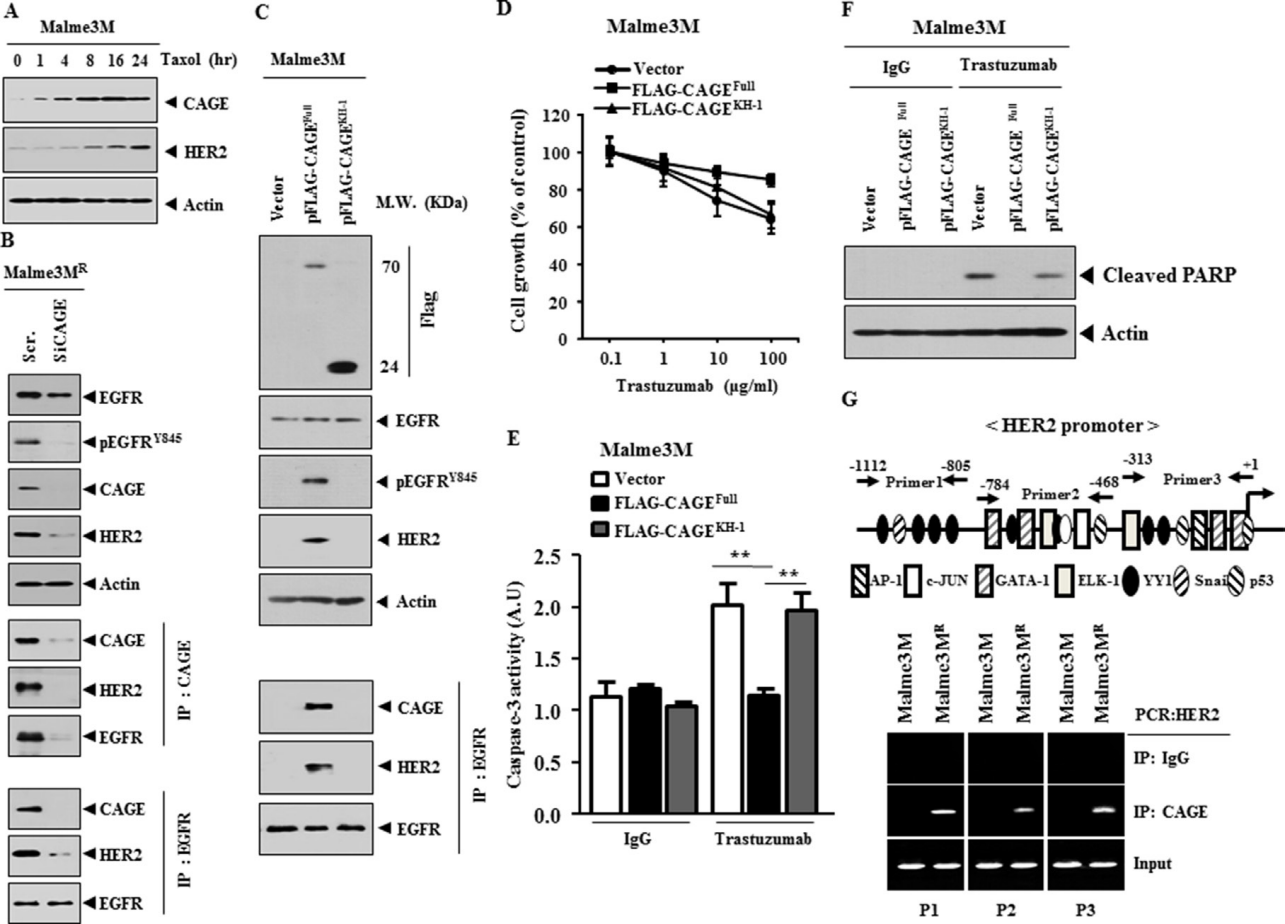
CAGE interacts with HER2, directly regulates the expression of HER2 and confers resistance to trastuzumab (**A**) Malme3M cells were treated with taxol (1 μM) for various time intervals. Cell lysates prepared at each time point were subjected to immunoblot analysis. (**B**) Malme3M^R^ cells were transfected with the indicated siRNA (10 nM). At 48 h after transfection, cell lysates prepared were immunoprecipitated with indicated antibody (2 μg/ml), followed by immunoblot analysis. Cell lysates were also subjected to immunoblot analysis. (**C**) Malme3M cells were transfected with the indicated construct (1 μg). At 48 h after transfection, cell lysates prepared were immunoprecipitated with indicated antibody (2 μg/ml), followed by immunoblot analysis. Cell lysates were also subjected to immunoblot analysis. (**D**) Malme3M cells were transiently transfected with the indicated construct (1 μg). At 24 h after transfection, cells were then treated with various concentrations of trastuzumab for 24 h, followed by MTT assays. (**E**) Malme3M cells were transiently transfected with the indicated construct (1 μg). At 24 h after transfection, cells were then treated with IgG (10 μg/ml) or trastuzumab (10 μg/ml) for 24 h, followed by caspase-3 activity assays. **p < 0.005. (**F**) Same as (E) except that immunoblot analysis was performed. (**G**) Shows the potential binding sites for transcriptional factors in the promoter sequences of HER2 (upper panel). ChIP assays were performed as described (lower panel).

Because EGFR regulated the response to trastuzumab (Figure 11A and 11B), we examined the relationship between CAGE and HER2. Taxol increased the expression of CAGE and HER2 (Figure 12A), suggesting that HER2 may confer resistance to taxol in association with CAGE. CAGE showed interactions with HER2 and EGFR in Malme3M^R^ cells (Figure 12B). Immunofluorescence staining showed lack of expression of CAGE and HER2 in Malme3M cells (Supplementary Figure S3A). CAGE showed co-localization with EGFR and HER2 in Malme3M^R^ cells (Supplementary Figure S3B). The down-regulation of CAGE decreased the expression of pEGFR^Y845^ and HER2 in Malme3M^R^ cells (Figure 12B). Malme3M^R^ cells transfected with siCAGE did not show the interaction of CAGE with EGFR or HER2 or the interaction between EGFR and HER2 (Figure 12B). Full-length CAGE, but not KH1 deletion construct, increased the expression of pEGFR^Y845^ and HER2, and induced interaction of EGFR with CAGE and HER2 (Figure 12C). These results suggest that EGFR and HER2 are necessary for anti-cancer drug-resistance conferred by CAGE. Full-length CAGE, but not KH1 deletion construct, conferred resistance to trastuzumab in Malme3M cells (Figure 12D), prevented caspase-3 activity from increasing in response to trastuzumab in Malme3M cells (Figure 12E) and prevented cleavage of PARP in response to trastuzumab in Malme3M cells (Figure 12F). HER2 promoter contains potential binding sites for HDAC2. CAGE, through interaction with HDAC2, confers resistance to anti-cancer drugs [42]. This led us to hypothesize that CAGE would bind to the promoter sequences of HER2. ChIP assays showed the binding of CAGE to the promoter sequences of HER2 (Figure 12G), suggesting that CAGE directly regulates the expression of HER2. Taken together, these results suggest that CAGE may confer resistance to trastuzumab through its interaction with and direct regulation of HER2.

### The down-regulation of CAGE decreases the tumorigenic potential of Malme3M^R^ cells and confers *in vivo* sensitivity to trastuzumab

Because CAGE conferred resistance to trastuzumab *in vitro* (Figure 12D and 12E), we examined the effect of CAGE on the *in vivo* response to trastuzumab. Malme3M^R^ cells showed *in vivo* resistance to trastuzumab (Supplementary Figure S4A). The down-regulation of CAGE decreased the tumorigenic potential of Malme3M^R^ cells and enhanced *in vivo* sensitivity of Malme3M^R^ cells to trastuzumab (Supplementary Figure S4A). Immunoblot analysis of tumor tissue lysates showed that the *in vivo* down-regulation of CAGE decreased the expression of pEGFR^Y845^ and HER2 (Supplementary Figure S4B). Immunoprecipitation of tumor tissue lysates showed that the *in vivo* down-regulation of CAGE prevented the interaction of CAGE with EGFR and HER2 and the interaction between EGFR and HER2 (Supplementary Figure S4B). Taken together, these results suggest that CAGE regulates the *in vivo* response to trastuzumab in a manner associated with its effect on the expression of pEGFR^Y845^, HER2 and the interaction of CAGE with EGFR and HER2.

### miR-217 regulates the expression of pEGFR^Y845^ and interactions of CAGE with EGFR and HER2

Malme3M^R^ cells showed higher expression level of CAGE and pEGFR^Y845^ than Malme3M cells (Supplementary Figure S5A). Malme3M^R-miR-217^ cells showed lower expression of CAGE and pEGFR^Y845^ than Malme3M^R^ cells (Supplementary Figure S5A). Unlike Malme3M^R^ cells, Malme3M^R-miR-217^ cells did not show the interaction between CAGE and EGFR (Supplementary Figure S5A). miR-217 inhibitor increased the expression of CAGE and pEGFR^Y845^ and induced the interaction between CAGE and EGFR in Malme3M cells (Supplementary Figure S5B). Malme3M^R –miR-217^ cells showed lower expression of CAGE, HER2 and pEGFR^Y845^ than Malme3M^R^ cells (Supplementary Figure S5C). Malme3M^R –miR-217^ cells did not show the interaction between CAGE and EGFR or the interaction between CAGE and HER2 (Supplementary Figure S5C). miR-217 inhibitor increased the expression of CAGE, pEGFR^Y845^ and HER2 in Malme3M^R-miR-217^ cells (Supplementary Figure S5C). miR-217 inhibitor also induced an interaction between CAGE and EGFR and an interaction between CAGE and HER2 in Malme3M^R-miR-217^ cells (Supplementary Figure S5C). miR-217 inhibitor targets CAGE to regulate the expression of pEGFR^Y845^ and HER2 and interactions of CAGE with EGFR and HER2 (Supplementary Figure S5D). Taken together, these results indicate that miR-217 regulates the response to EGFR inhibitors and trastuzumab through its effect on the interactions among CAGE, EGR and HER2.

### HER2 is necessary for an interaction between CAGE and EGFR, and regulates the response to anti-cancer drugs

**Figure 13:**
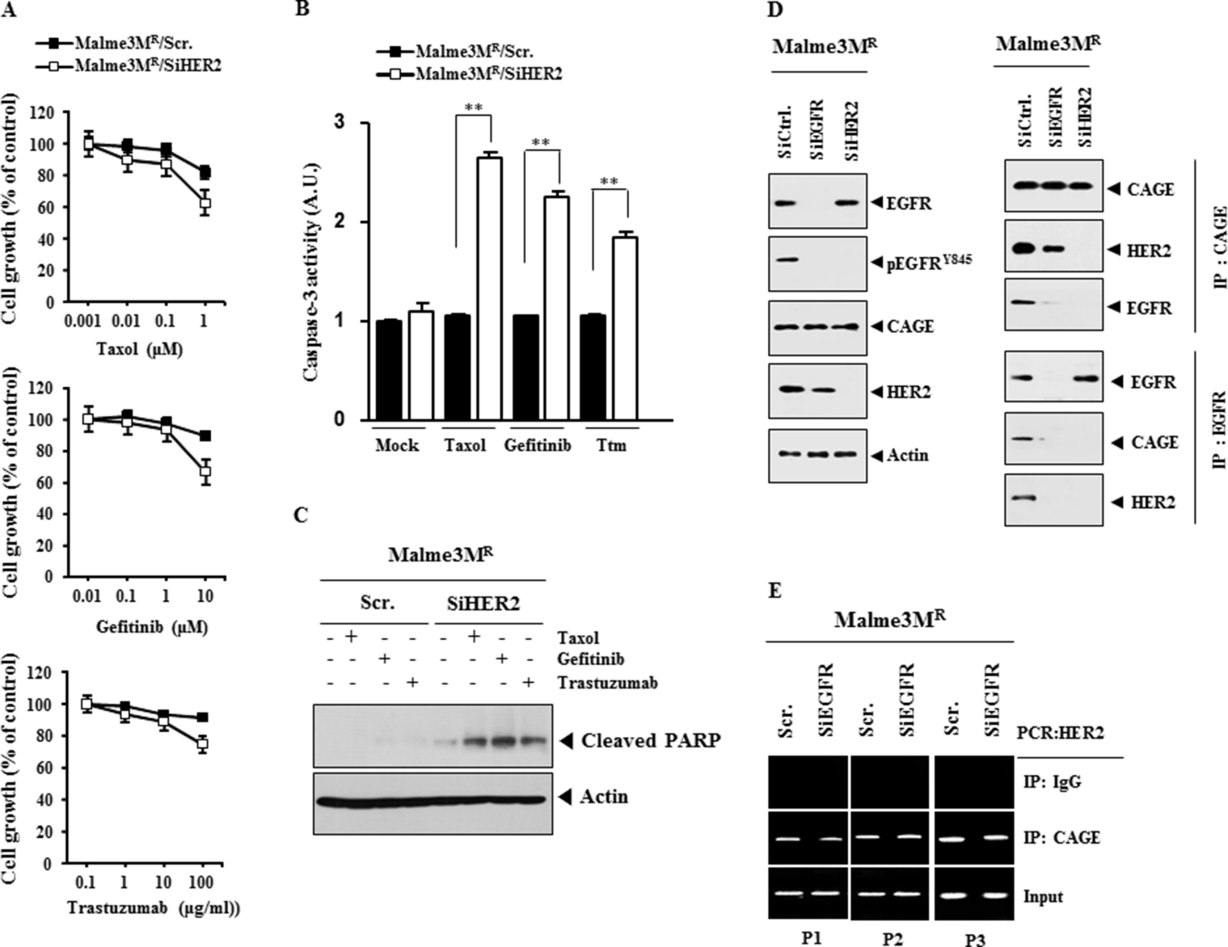
HER2 is necessary for the interaction between CAGE and EGFR, and regulates the response to anti-cancer drugs (**A**) Malme3M^R^ cells were transiently transfected with the indicated siRNA (each at 10 nM). At 24 h after transfection, cells were then treated with various concentrations of taxol, gefitinib or trastuzumab for 24 h, followed by MTT assays. (**B**) Malme3M^R^ cells were transiently transfected with the indicated siRNA (10 nM). At 24 h after transfection, cells were then treated with various concentrations of taxol (1 μM), gefitinib (1 μM) or trastuzumab (10 μg/ml) for 24 h, followed by caspase-3 activity assays. **p < 0.005. Ttm denotes trastuzumab. (**C**) Same as (B) except that immunoblot blot analysis was performed. (**D**) Malme3M^R^ cells were transfected with the indicated siRNA (10 nM). At 48 h after transfection, cell lysates were subjected to immunoblot and immunoprecipitation analysis. (**E**) Malme3M^R^ cells were transfected with the indicated siRNA (10 nM). At 48 h after transfection, cell lysates were subjected to ChIP assays.

The down-regulation of HER2 enhanced the sensitivity to taxol, gefitinib and trastuzumab (Figure 13A), increased caspase-3 activity (Figure 13B) and PARP cleavage (Figure 13C) in response to these anti-cancer drugs. The down-regulation of HER2 decreased the expression of pEGFR^Y845^, but not CAGE or EGFR (Figure 13D), and prevented the interaction between CAGE and EGFR (Figure 13D). The down-regulation of EGFR did not affect the interaction between CAGE and HER2 (Figure 13D) or the binding of CAGE to the promoter sequences of HER2 (Figure 13E). Taken together, these results suggest that HER2 may confer resistance to anti-cancer drugs by regulating the activation of EGFR and the interaction between CAGE and EGFR.

### The inactivation of EGFR confers sensitivity to anti-cancer drugs and inhibits interactions of EGFR with CAGE and HER2

**Figure 14:**
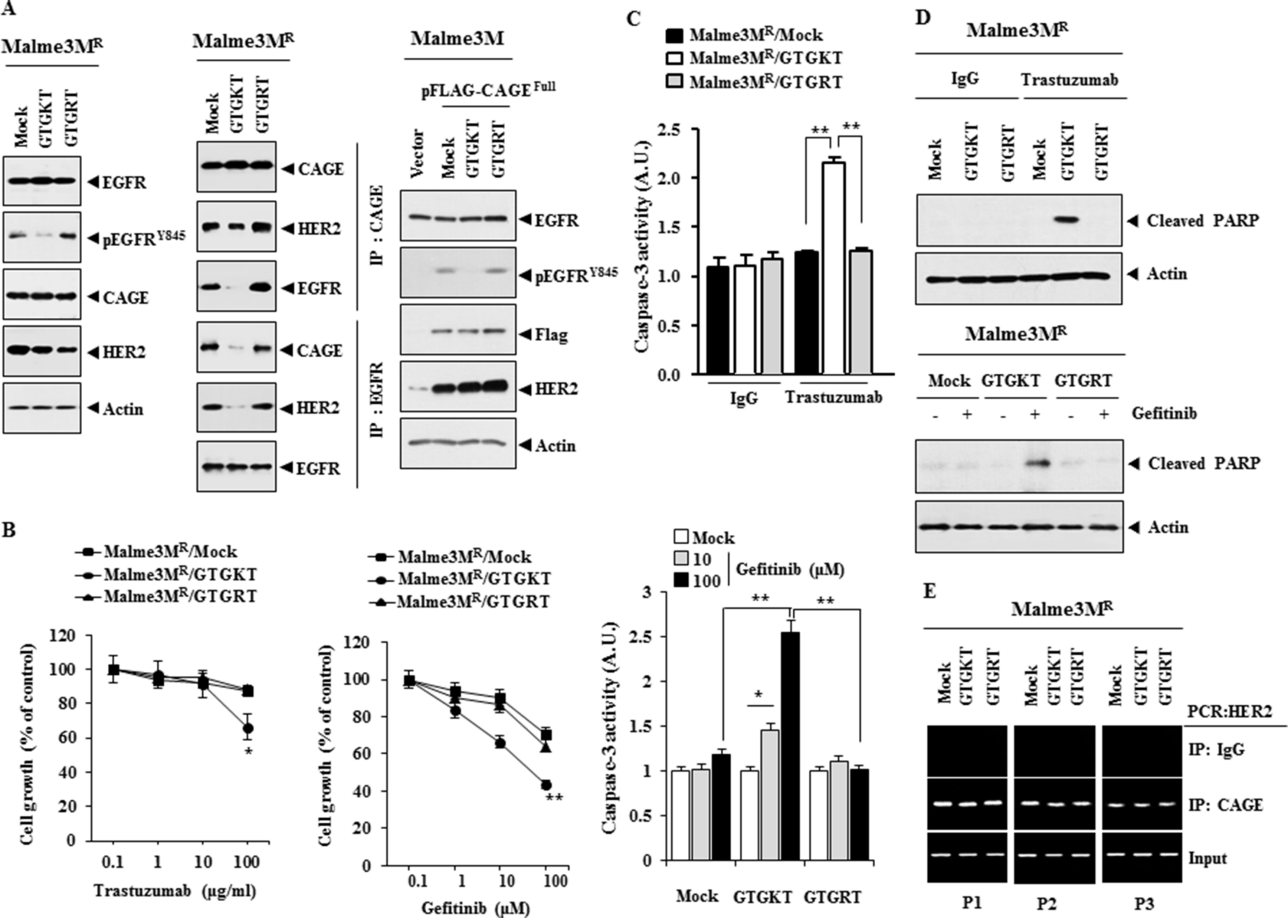
The inactivation of EGFR confers sensitivity to anti-cancer drugs and inhibits interactions of EGFR with CAGE and HER2 (**A**) Malme3M^R^ cells were treated with the indicated peptide (each at 10 μM) for 48 h. Cell lysates were then isolated and subjected to immunoprecipitation and immunoblot analysis (left panel). Malme3M cells were transfected with the indicated construct (each at 1 μg) along with the indicated peptide (10 μM). At 48 h after transfection, cell lysates were subjected to immunoblot analysis (right panel). (**B**) Malme3M^R^ cells were treated with the indicated peptide (each at 10 μM) for 48 h. Cells were then treated with various concentrations of trastuzumab or gefitnib for 24 h, followed by MTT assays. *p < 0.05; **p < 0.005. (**C**) Malme3M^R^ cells were treated with the indicated peptide (each at 10 μM) for 48 h. Cells were then treated with IgG (100 μg/ml), trastuzumab (100 μg/ml) or gefitinib (10, 100 μM) for 24 h, followed by caspase-3 activity assays. *p < 0.05; **p < 0.005. (**D**) Malme3M^R^ cells were treated with the indicated peptide (each at 10 μM) for 48 h. Cells were then treated with IgG (100 μg/ml), trastuzumab (100 μg/ml) or gefitinib (10 μM) for 24 h, followed by immunoblot analysis. (**E**) Same as (A) except that ChIP assays were performed.

CAGE-derived ^269^GTGKT^273^ peptide binds to CAGE and confers sensitivity to celastrol and taxol in Malme3M^R^ cells (personal observations). GTGKT corresponds to the ATP-binding site within the DEAD box domain of CAGE. Immunoprecipitation of biotin-labeled GTGKT showed the binding of GTGKT peptide to CAGE (personal observations). This implies that GTGKT may prevent interaction of CAGE with EGFR and/or HER2. GTGKT peptide displays tumor homing potential (personal observations). We investigated the effect of the inactivation of EGFR on the interaction between CAGE and EGFR and the interaction between EGFR and HER2. GTGKT, but not GTGRT peptide, decreased the expression of pEGFR^Y845^ in Malme3M^R^ cells (Figure 14A). However, GTGKT peptide did not affect the expression of CAGE or HER2 in Malme3M^R^ cells (Figure 14A). GTGKT peptide inhibited the interaction between CAGE and EGFR and inhibited the interaction between EGFR and HER2 in Malme3M^R^ cells (Figure 14A). However, GTGKT peptide did not affect the interaction between CAGE and HER2 (Figure 14A). This implies that the domain of CAGE necessary for binding to EGFR is different from that of CAGE necessary for binding to HER2. GTGKT peptide decreased the expression of pEGFR^Y845^ in Malme3M cells transfected with CAGE (Figure 14A). GTGKT peptide inhibited the interaction between CAGE and EGFR and inhibited the interaction between EGFR and HER2 in Malme3M cells transfected with CAGE (data not shown). GTGKT peptide, but not GTGRT, enhanced the sensitivity to trastuzumab and gefitinib (Figure 14B). GTGKT peptide enhanced caspase-3 activity in response to trastuzumab and gefitinib in Malme3M^R^ cells (Figure 14C) and induced cleavage of PARP in response to trastuzumab and gefitinib in Malme3M^R^ cells (Figure 14D). GTGKT peptide did not affect the binding of CAGE to the promoter sequences of HER2 (Figure 14E). Taken together, these results suggest that EGFR mediates anti-cancer drug-resistance conferred by CAGE through its effect on the interaction of EGFR with CAGE and HER2.

## DISCUSSION

Malme3M^R^ cells show lower expression level of miR-217 and miR-335 while displaying higher expression level of miR-326 than Malme3M cells (Figure 1A). miR-326 forms a negative feedback loop with HDAC3 and regulates the response to anti-cancer drugs (21). In this study, we found that HDAC3 functions upstream of CAGE and directly regulates the expression of CAGE (personal observations). It is probable that miR-326-HDAC3 negative feedback loop regulates the expression of CAGE. It would be necessary to examine the effect of miR-326 on the expression of transcriptional factors that bind to the promoter sequences of CAGE. CAGE promoter contains potential binding sites for DNMT1, Sp1, GATA-1 and Elk-1 (personal observations). DNMT1 negatively regulates the expression of CAGE [42]. It is probable that miR-326 may increase the expression of CAGE by regulating the expression of HDAC3 and transcriptional factor such as Sp1. We reported that miR-335 increases the expression of HDAC3 by negatively regulating the expression of SIAH2 [48]. Therefore miR-335 may negatively regulate the expression of CAGE. It would be interesting to examine the effect of mR-335 on the expression of DNMT1, Sp1, GATA-1 and Elk-1. For better understanding mechanism of anti-cancer drug-resistance, it would be necessary to identify miRNAs that are regulated by CAGE.

ChIP assays show the binding of CAGE to the site 2 of the miR-217 promoter sequences (Figure 4C). Site 2 of the miR-217 promoter sequence contains the binding sites for Sp1, YY1, AP1 and GATA1 (Figure 4A). In this study, we found the interaction between CAGE and Sp1 in Malme3M^R^ cells (personal observations). Malme3M^R^ cells show higher expression of Sp1 than Malme3M cells (personal observations). Sp1, a target of miR-145, confers resistance to paclitaxel *in vitro* and *in vivo* [49]. It is probable that CAGE, through interaction with Sp1, binds to the promoter sequences of miR-217 to regulate the expression of miR-217. CAGE increases the expression of cyclin D1 in an AP1 and E2F-dependent manner [41]. It is probable that Sp1 may increase the expression of cyclin D1. It is probable that miR-217 negatively regulates the expression of cyclin D1. miR-217 negatively regulates the tumorigenic potential (Figure 7A) and the angiogenic potential of cancer cells (Figure 7C and 7D). We previously reported that recombinant CAGE protein enhances the angiogenic potential of cancer cells [43]. It would be necessary to examine the effect of miR-217 on the expression of angiogenic factors. miR-217 inhibitor promotes epithelial to mesenchymal transition (EMT) and down-regulated miR-217 is positively correlated with late tumor stage, lymphatic invasion, vascular infiltration and distant metastasis [50]. We show that miR-217 negatively regulates the metastatic potential of cancer cells (Supplementary Figure 1A and 1C). It would be necessary to examine the effect of miR-217 on factors that regulate the metastatic potential of cancer cells. This would be helpful for better understanding of the mechanism of anti-cancer drug-resistance conferred by CAGE.

Taxol increases the phosphorylation of EGFR [51–53]. EGFR inhibition enhances sensitivity to taxol in prostate cancer cells [54]. PD168393, an inhibitor of EGFR, potentiates cytotoxic effect of taxol against prostate cancer cells [54]. These reports suggest that taxol-resistance is related with the resistance to EGFR inhibitors. Taxol increases the expression of pEGFR^Y845^ in Malme3M cells (Figure 9A). CAGE increases the phosphorylation of EGFR, but not the expression of EGFR (Figure 10B). CAGE confers resistance to taxol, gefitinib and cetuximab (Figure 10D). Src kinase inhibition enhances antitumor activity of taxol in ovarian cancer and taxol increases the expression of pSrc [55]. It is therefore probable that src is responsible for the activation of EGFR. It would be necessary to examine the effect of Src on the phosphorylation of EGFR by CAGE.

EGFR interacts with HER2 [56] and high-level expression of EGFR attenuates the effect of anti-HER2-directed antibodies in HER2-amplified breast cancer cells [57]. This implies that the resistance to EGFR inhibitors is closely related with the resistance to HER2 inhibitor such as trastuzumab. We show that the down-regulation of EGFR enhances sensitivity to trastuzumab (Figure 11A). Taxol increases the expression of HER2 as well as CAGE (Figure 12A) and the expression of HER2 is regulated by CAGE in Malme3M^R^ cells (Figure 12B and 12G). HER2 promoter contains binding site for p53 (Figure 12B). P53 negatively regulates the expression of CAGE (42). It is probable that p53 negatively regulates the expression of HER2 by binding to the promoter sequences of HER2. HER2 interacts with CAGE and EGFR in Malme3M^R^ cells (Figure 12B). HER2 regulates the expression of pEGFR^Y845^ (Figure 13D). CDCP1 binds to HER2 through its intracellular domain, thereby increasing HER2 interaction with the non-receptor tyrosine kinase c-Src, leading to trastuzumab resistance [58]. Src kinase pathway is involved in trastuzumab–resistance in HER2-amplified breast cancers [59]. EGFR and HER2 are transactivated by c-Src and MMPs [60]. It is probable that HER2, through interaction with src, regulates the expression of pEGFR^Y845^. Given the fact that CAGE interacts with HER2, it is probable that CAGE interacts with src. miR-7 reverses trastuzumab resistance through its effect on EGFR and Src kinase [61]. It would be interesting to examine the effect of miR-7 on the expression of CAGE and miR-217. It is probable that miR-7 may negatively regulate the expression of CAGE.

CAGE-binding ^269^GTGKT^273^ peptide enhances the sensitivity to taxol in Malme3M^R^ cells (personal observations). GTGKT peptide decreases the expression of pEGFR^Y845^ (Figure 14A). It would be interesting to examine the effect of GTGKT on src activity. GTGKT peptide may inhibit the interaction between HER2 and src. For better understanding of the effect of GTGKT peptide on anti-cancer drug-resistance, it will be necessary to identify binding sites for GTGKT on CAGE. Because CAGE regulates the expression of pEGFR^Y845^ (Figure 12B), it is probable that CAGE displays kinase activity. If so, it will be necessary to examine the effect of GTGKT peptide on kinase activity of CAGE. Because GTGKT peptide enhances sensitivity to gefitinib and trastuzumab (Figure 14B), it is probable that GTGKT peptide may negatively regulate the tumorigenic and metastatic potential of Malme3M^R^ cells.

Notch-1 is associated with the resistance to EGFR tyrosine kinase inhibitors [62]. Trastuzumab increases Notch activity, which leads to resistance to trastuzumab [63]. Notch directly binds to the cyclin D1 promoter sequences to induce the expression of cyclin D1 [64]. Malme3M^R^ cells express higher expression level of Notch-1 than Malme3M cells (personal observations). It is probable that Notch signaling regulates the expression of CAGE and the response to EGFR inhibitors and HER2 inhibitors. It would be necessary to identify miRNAs that regulate the expression of Notch-1 for better understanding the mechanism of anti-cancer drug-resistance conferred by CAGE.

TargetScan analysis predicts the binding of mR-495,-329,-338, and -362–3p to the 3′-UTR of HER2. miR-338–3p inhibits proliferation by regulating cyclinD1, and HBx down-regulates miR-338–3p in hepatocellular carcinoma [65]. It will be necessary to examine the effect of miR-338–3p on the expression of CAGE, miR-217 and HER2. It would be interesting to examine the effect of miR-338–3p on the expression of transcriptional factors such as Sp1, YY1, AP1 and GATA-1. miR-362–3p reduces cell viability, and proliferation mainly due to cell cycle arrest [65]. E2F1 serves as a target of miR-362–3p [66]. CyclinD1 expression is dependent on E2F1 [41]. It is therefore probable that miR-362–3p negatively regulates the expression of CAGE. ChIP assays show the binding of CAGE to miR-362–3p promoter sequences (personal observations), suggesting a negative feedback loop between CAGE and miR-362–3p. It is probable that miR-362–3p may regulate the response to gefitinib and trastuzumab. Over-expression of miR-495 in glioma cells downregulates the expression of cyclin-dependent kinase 6 (CDK6) and inhibits retinoblastoma phosphorylation, and knockdown of CDK6 results in cell cycle arrest at the G1/S transition and inhibition of cell proliferation [67]. miR-495 targets the 3′-UTR of the MDR1 gene and reduces expression of the MDR1 gene [68]. This suggests the role of miR-495 in the response to anti-cancer drugs. It would be necessary to examine the effect of miR-495 on the expression of pEGFR^Y845^, interactions of CAGE with EGFR and HER2. The identification of more targets of these miRNAs will be necessary for better understanding the mechanism of anti-cancer drug-resistance conferred by CAGE.

In this study, we show that miR-217-CAGE feedback loop regulates the response to various anti-cancer drugs, such as taxol, gefitinib, cetuximab and trastuzumab, through regulation of EGFR activation and CAGE interactions with EGFR and HER2. The effect of CAGE on the phosphorylation of EGFR and on the interaction between EGFR and HER2 has never been reported. The effect of CAGE on the response to EGFR inhibitor and HER2 has not been reported, either. miR-217-CAGE loop serves as a target for the overcoming resistance to EGFR inhibitors and HER2 inhibitors in melanoma patients.

## MATERIALS AND METHODS

### Materials

Anti mouse and anti rabbit IgG-horse radish peroxidase conjugate antibodies were purchased from Pierce Company. An ECL (enhanced chemiluminiscence) kit was purchased from Amersham. Lipofectamin and Plus^™^ reagent were purchased from Invitrogen (Carlsbad, CA, USA). miRNA array kit was purchased from Signosis, Inc. miR mimic, miR inhibitor and siRNAs were purchased from Bioneer Co. (Daejon, Korea). CAGE-derived peptides were synthesized by Peptron Company (Daejeon, Korea), with the sequences GTGKT and GTGRT and a purity level > 95%.

### Cell lines and cell culture

Cancer cell lines (SNU387^R^, Malme3M^R^ and AGS^R^) made resistant to celastrol, a microtubule-targeting drug were established by stepwise addition of celastrol. Cells surviving drug treatment (attached fraction) were obtained and used throughout this study. Malme3M^R^ or SNU387^R^ cells that stably express miR-217 (Malme3M^R-miR-217^ or SNU387^R-miR-217^) were also selected by G418 (400 μg/ml). Malme3M^R^ cells that stably express anti-sense CAGE (Malme3M^R- As-CAGE^) were also selected by G418. Taxol-resistant cancer cell lines (Malme3M^R-Taxol^) were made by stepwise addition of taxol. Cancer Cells were grown in Dulbecco's modified Eagle's medium containing heat-inactivated fetal bovine serum, 2 mM L-glutamine, 100 units/ml penicillin, and 100 μg/ml streptomycin (Invitrogen). Cultures were maintained in 5% CO_2_ at 37°C. Human umbilical vein endothelial cells (HUVECs) were isolated from human umbilical cord veins according to the standard procedures [43].

### Cell viability determination

The cells were assayed for their growth activity using the 3-(4, 5-dimethylthiazol-2-yl)-2, 5-diphenyltetrazolium bromide (MTT; Sigma). Viable cell number counting was carried out by trypan blue exclusion assays.

### Caspase-3 activity assays

Caspase-3 activity was measured according to the manufacturer's instructions (BioVision, Palo Alto, CA). Cells were lysed in 0.1 M HEPES buffer, pH 7.4, containing 2 mM dithiothreitol, 0.1% CHAPS, and 1% sucrose. Cell lysates were incubated with a colorimetric substrate, 200 μM Ac-DEVD-*p*-nitroanilide, for 30 min at 30°C. The fluorescence was measured at 405 nm using a microtiter plate reader.

### Immunoblot and immunoprecipitation analysis

Immunoblot analysis and immunoprecipitation were performed according to the standard procedures [21]. For analysis of proteins from tumor tissues, frozen samples were grounded to a fine powder using a mortar and pestle over liquid nitrogen. Proteins were solubilized in RIPA buffer containing protease inhibitors and insoluble material removed by centrifugation.

### miRNA array analysis

miRNA analysis was performed according to the standard procedures [43]. The miRNA array kit was purchased from Koma Biotech (Seoul, Korea).

### miRNA target analysis

Genes that contain the miR-binding site(s) in the UTR were obtained using the TargetScan program.

### Chemo invasion assays

The invasive potential of cancer cells was determined by using a transwell chamber system with 8-μm pore polycarbonate filter inserts (CoSTAR, Acton, MA). The lower and upper sides of the filter were coated with gelatin and Matrigel, respectively. Trypsinized cells (5 × 10^3^) in the serum-free RPMI 1640 medium containing 0.1% bovine serum albumin were added to each upper chamber of the transwell. RPMI 1640 medium supplemented with 10% fetal bovine serum was placed in the lower chamber and cells were incubated at 37°C for 16 h. The cells were fixed with methanol and the invaded cells were stained and counted. Results were analyzed for statistical significance using the Student's *t* test. Differences were considered significant when *p* < 0.05.

### Wound migration

Cells were plated overnight to achieve a confluent layer in 24-well plates. A scratch was made on the cell layer with a micropipette tip and cultures were washed twice with serum-free medium. Cells were then transfected with construct of interest. Wound healing was visualized by comparing photographs taken at the time of transfection and 48 h later.

### RNA extraction and quantitative real-time PCR (qRT-PCR)

Total miRNA was isolated using the *mir*Vana miRNA isolation kit (Ambion). miRNA was extended by a poly (A) tailing reaction using the A-Plus Poly (A) Polymerase Tailing Kit (Cell Script). cDNA was synthesized from miRNA with poly (A) tail using a poly (T) adaptor primer and qScript^™^ reverse transcriptase (Quanta Biogenesis). The expression level of miR-217 or other miRNA gene was quantified with SYBR Green qRT-PCR kit (Ambion) using a miRNA-specific forward primer and a universal poly (T) adaptor reverse primer. The expression of miR-217 was defined based on the threshold (Ct), and the relative expression levels were calculates as 2^− [(Ct of miR-217)-(Ct of U6)]^ after normalization with reference to the expression of U6 small nuclear RNA. For quantitative PCR, SYBR PCR Master Mix (Applied Biosystems) was used in a CFX96 Real-Time System thermocycler (BioRad).

### pGL3–3′ UTR-CAGE

To generate the pGL3–3′-UTR-CAGE construct, a 136-bp human CAGE gene segment encompassing 3′-UTR was PCR amplified and subcloned into the XbaI site of pGL3 luciferase plasmid. The mutant pGL3–3′-UTR-CAGE construct was made with the QuikChange site-directed mutagenesis kit (Stratagene). Luciferase activity assay was performed according to the instruction manual (Promega).

### Chromatin immunoprecipitation (ChIP) assays

Assays were performed according to manufacturer's instruction (Upstate). For detection of binding of protein of interest to miR-217 promoter sequences, specific primers of miR-217 promoter-1 sequences [5′-GTAATATAATAAACAAGAAAACTTTTGGAAGTG-3′ (sense) and 5′-GTTTTCCTCCCTGCCAGCTTT ATT-3′ (antisense)], miR-217 promoter-2 sequences [5′-ACC CCATCTCTACTAAAAATACAAAAATTAG-3′ (sense) and 5′-TATATCTAACCTACTAATCAAGCCACCTT AGCT-3′ (antisense)] and miR-217 promoter-3 sequences [5′-AGT AGGTTAGATA TAGAATTTTA AAAAGCTA TTTTT-3′ (sense) and 5′-CCCAATTTACCAAGAGAG ATATATTACAATATAA-3′ (antisense)] were used. For detection of binding of protein of interest to HER2 promoter sequences, specific primers of HER2 promoter-1 sequences [5′-TGTTAGCCAGGATGGTCTCG-3′ (sense) and 5′-CCCATCTCCCACACCTCTTT-3′ (antisense)], HER2 promoter-2 sequences [5′-TGCCTTTGATCCCTTC TTGA-3′ (sense) and 5′-GGTTTCTTCTTTGCCCCT TG-3′ (antisense)] and HER2 promoter-3 sequences [5′-GGGAGTTCAAGACCAGCCTC-3′ (sense) and 5′-GGGGCATATCTTCTGGAATCTT-3′ (antisense)] were used.

### Transfection

All transfections were performed according to the manufacturer's instructions. Lipofectamine and Plus reagents (Invitrogen) were used. The construction of siRNA was carried out according to the instruction manual provided by the manufacturer (Ambion, Austin, TX). For miR-217 knockdown, cells were transfected with 50 nM of oligonucleotide (inhibitor) with Lipofectamine 2000 (Invitrogen), according to the manufacturer's protocol. The sequences used were: 5′-UACUGCAUCAGGAACUGAUUGGA-3′ (miR-217 inhibitor); and 5′-GCCUCCGGCUUCGCACCUCU-3′ (control inhibitor).

### Immunofluorescence staining

Cells were seeded onto glass coverslips in 24-well plates and fixed with 4% paraformaldehyde (v/v) for 10 min and then permeabilized with 0.4% Triton X-100 for 10 min. Nonspecific antibody binding sites were blocked by incubation with 1% BSA in TBST for 30 min. Cells were then incubated with primary antibody specific to CAGE (1:200; BD Biosciences), HER2 (1:200; Santa Cruz), EGFR (1:200; BD Biosciences) or pEGFR^Y845^ (1:200; Santa Cruz) for 2 h, followed by washing with TBS-T three times. Anti-goat IgG-FITC (for detection of pEGFR^Y845^, EGFR and HER2) or anti-rabbit Alexa Fluor 586 (for detection of CAGE) secondary antibody (Molecular Probes) was added to cells and incubated for 1 h. Cover slips were then washed and mounted by applying Mount solution (Biomeda, Foster City, CA). Fluorescence images were acquired using a confocal laser scanning microscope and software (Fluoview version 2.0) with × 60 objective (Olympus FV300, Tokyo, Japan).

### Immunohistochemistry

Paraffin-embedded tissue sections were immunostained using the Vecta stain ABC Elite Kit (Vector Laboratories). Tissue sections were deparaffinized with xylene and washed in ethanol. Endogenous peroxidase activity is blocked with 3% hydrogen peroxide and H_2_O for 10min. Slides were then blocked with 5% normal goat serum in TBS containing 0.1% Tween-20 (TBS-T) for 1 h. For immunohistochemistry, a primary antibody to CAGE (1:100, Santa Cruz), MDR1 (1:100, Santa Cruz), EGFR (1:100, Santa Cruz), pEGFR ^Y845^ (1:100, Santa Cruz) or IgG (1:100, Santa Cruz) was added and incubation continued at 4°C for 24 h. After washing with TBS-T, slides were treated with biotinylated secondary antibody for 30 min. After washing, slides were incubated in the ABC complex for 30 min, and then stained with diaminobenzidine (DAB, Sigma). For H & E staining, tumor tissue samples were fixed in 10% (v/v) buffered formalin, embedded in paraffin, sectioned at 4 μm, and then stained with hematoxylin and eosin. Sections were mounted using Fixo gum rubber cement (Mercateo, München, Germany).

### Tumorigenic potential

Athymic nude mice (BALB/c nu/nu, 5–6-week-old females) were obtained from Orient Bio Inc. (Seoul, Korea) and were maintained in a laminar air-flow cabinet under aseptic conditions. All animal experiments were approved by the Institutional Animal Care and Use Committee of Kangwon National University (KW-140707–1). Cancer cells (1 × 10^6^) were injected subcutaneously into the dorsal flank area of the mice. Tumor volume was determined by direct measurement with calipers and calculated by the following formula: length × width × height × 0.5. To determine the effect of miR-217 inhibitor on *in vivo* tumorigenic potential of cancer cells, control inhibitor (40 μg/kg or 50 μM/kg) or miR-217 inhibitor (40 μg/kg or 50 μM/kg) was injected following the establishment of sizable tumor by Malme3M cells, via tail vein five times in a total of 30 days. To compare the tumorigenic potential of Malme3M^R^ and Malme3M^R-miR-217^ cells, Malme3M^R^ or Malme3M^R-miR-217^ cells (1 × 10^6^) were injected subcutaneously into the dorsal flank area of the mice. To determine the effect of CAGE on the *in vivo* resistance to trastuzumab, scrambled siRNA (100 nM) or CAGE siRNA (100 nM) was injected along with or without trastuzumab (10 mg/kg), following the establishment of sizable tumor by Malme3M^R^ cells, via tail vein 4 times in a total of 25 days.

### *In vivo* metastasis assay

Female athymic nude mice were used for the studies. Malme3M Cells (10^6^ cells in PBS) were injected intravenously into the tail vein of 4-week old athymic nude mice, and the extent of lung metastasis was evaluated. Control inhibitor (50 μM/kg) or *miR-217* inhibitor (50 μM/kg) was injected intravenously into the tail vein of athymic nude mice five times. After 4 weeks, the mice were sacrificed and analyzed for the lung colonization. The lungs were rinsed with PBS and then fixed and stained with Bouin›s solution. After 24 h, the lungs were rinsed in water to remove excess Bouin's solution and the extent of lung metastases was quantified. To determine the effect of *miR-217* mimic on the metastatic potential of cancer cells, Malme3M^R^ or Malme3M^R-miR-217^ cells (10^6^ cells in PBS) were injected intravenously into the tail vein of 4-week old athymic nude mice. Control mimic (50 μM/kg) or miR-217 mimic (50 μM/kg) was injected intravenously into the tail vein of athymic nude mice five times. After 4 weeks, the mice were sacrificed and analyzed for the lung colonization.

### *In vivo* matrigel plug assay

Seven week-old BALB/C mice (DBL Co., Ltd, Korea) were injected subcutaneously with 0.1 ml of matrigel containing the conditioned medium and 10 units of heparin (Sigma). The injected matrigel rapidly formed a single, solid gel plug. After 8 days, the skin of the mouse was easily pulled back to expose the matrigel plug, which remained intact. Hemoglobin (Hb) content in the matrigel plugs was measured using the Drabkin reagent (Sigma, USA) for quantification of blood vessel formation.

### Intravital microscopy

Male BALB/c mice (6–8 week old) were obtained from Daehan Biolink (Korea). *In vivo* angiogenesis was assessed as follows. The mice were anesthetized with 2.5% avertin (v/v) via intraperitoneal injection (Surgivet, USA), and abdominal wall windows were implanted. Next, a titanium circular mount with eight holes on the edge was inserted between the skin and the abdominal wall. Growth factor-reduced matrigel containing the conditioned medium was applied to the space between the windows, and a circular glass cover slip was placed on top and fixed with a snap ring. After four days, the animals were anesthetized and injected intravenously with 50 μl of 25 ng/ml fluorescein isothiocyanate-labeled dextran (molecular weight, Mr ∼2,000,000) via the tail vein. The mice were then placed on a Zeiss Axiovert 200 M microscope. The epi-illumination microscopy setup included a 100-W mercury lamp and filter set for blue light. Fluorescence images were recorded at random locations of each window using an electron-multiplying charge coupled device camera (Photo Max 512, Princeton Instruments, USA) and digitalized for subsequent analysis using the Metamorph program (Universal Imaging, USA). The assay was scored from 0 (negative) to 5 (most positive) in a double-blinded manner.

### Endothelial cell tube formation assays

Growth factor–reduced matrigel was pipetted into pre-chilled 24-well plates (200 μl matrigel per well) and polymerized for 30 min at 37°C. The HUVECs were placed onto the layer of matrigel in 1 ml of M199 containing 1% FBS. After 6 to 8 h of incubation at 37°C in a 95%:5% (v/v) mixture of air and CO_2_, the endothelial cells were photographed using an inverted microscope (magnification, × 100; Olympus). Tube formation was observed using an inverted phase contrast microscope. Images were captured with a video graphic system. The degree of tube formation was quantified by measuring the length of tubes in five randomly chosen low-power fields (× 100) from each well using the Image-Pro plus v4.5 (Media Cybernetics, San Diego, CA, USA).

### Statistical analysis

Statistical differences were determined by using the Student's *t* test.

## SUPPLEMENTARY MATERIALS FIGURES


