# Supplementary material for: miR-217 and CAGE form feedback loop and regulates the response to anti-cancer drugs through EGFR and HER2
Source: Oncotarget. 2016 Feb 4;7(9):10297–321. doi: 10.18632/oncotarget.7185 (PMC4891121; doi:10.18632/oncotarget.7185)
Supplement: Supplementary file 1 [file oncotarget-07-10297-s001.pdf]

miR-217 and CAGE form feedback loop and regulates the response to anti-cancer drugs through EGFR and HER2

Supplementary Materials

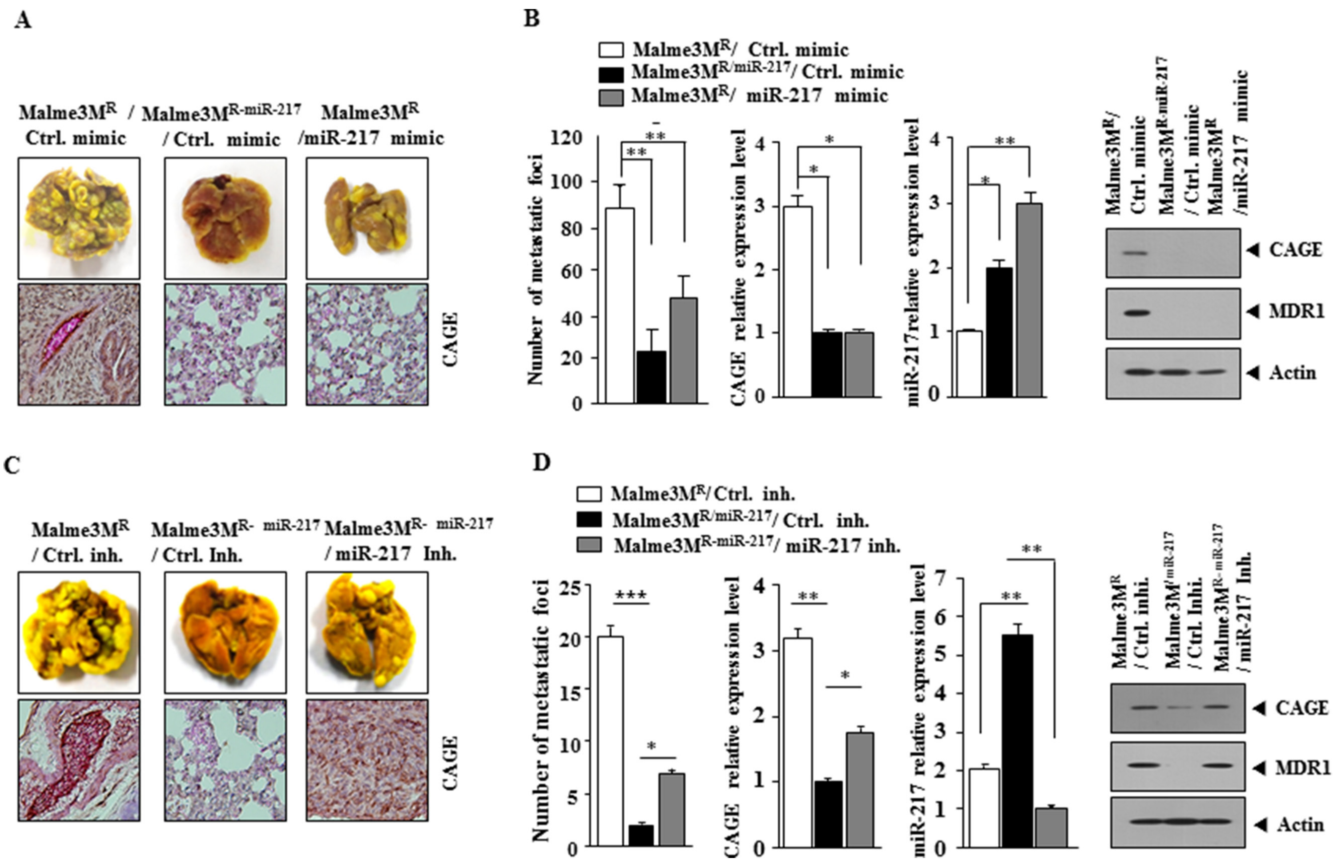

Supplementary Figure S1: miR-217 negatively regulates the metastatic potential of Malme3M^{R} cells. (**A**) Each experimental group consists of five athymic nude mice. Each figure shows a representative image of the mice in each experimental group. Control mimic (50 µM/kg) or miR-217mimic (50 µM/kg) was intravenously injected five times over a total of 4 weeks. Arrows show the metastatic foci. The extent of lung metastases was quantified by staining with Bouin's solution. Immunohistochemistry staining employing lung tumor tissue was performed as described. (**B**) QRT-PCR analysis employing lung tumor tissue was performed to determine the expression of CAGE and miR-217. Lung tumor tissue lysates were also subjected to immunoblot analysis. *$p < 0.05$; **$p < 0.005$. (**C**) Same as (A) except that control inhibitor (50 µM/kg) or miR-217 inhibitor (50 µM/kg) was intravenously injected five times over a total of 4 weeks. Immunohistochemistry staining employing lung tumor tissue was performed as described. (**D**) Lung tumor lysates were isolated and qRT-PCR analysis was performed to determine the expression of CAGE and miR-217. Lung tumor tissue lysates were also subjected to immunoblot analysis. *$p < 0.05$; **$p < 0.005$; ***$p < 0.0005$.

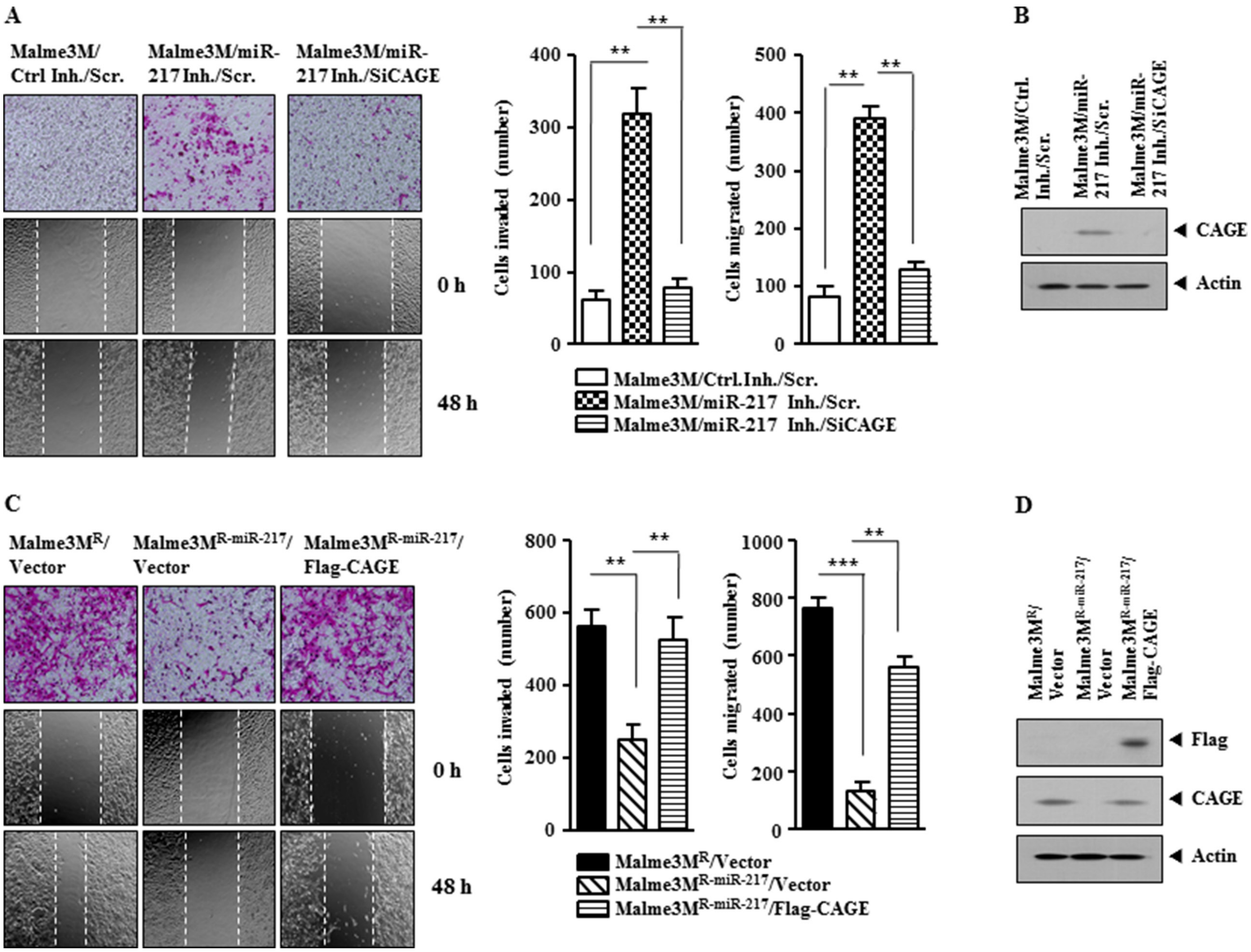

Supplementary Figure S2: The effect of miR-217 on the migration and invasion potential of cancer cells is dependent on CAGE. (**A**) Malme3M cells were transiently transfected with the indicated inhibitor (10 nM) along with the indicated siRNA (10 nM). At 48 h after transfection, cells were subjected to wound migration assays or invasion assays. $^{**}p < 0.005$. (**B**) Same as (A) except that immunoblot analysis was performed. (**C**) The indicated cancer cells were transfected with control vector (1 μg) or Flag-CAGE (1 μg). At 48 h after transfection, cells were subjected to wound migration assays or invasion assays. $^{**}p < 0.005$; $^{***}p < 0.0005$. (**D**) Same as (C) except that immunoblot analysis was performed.

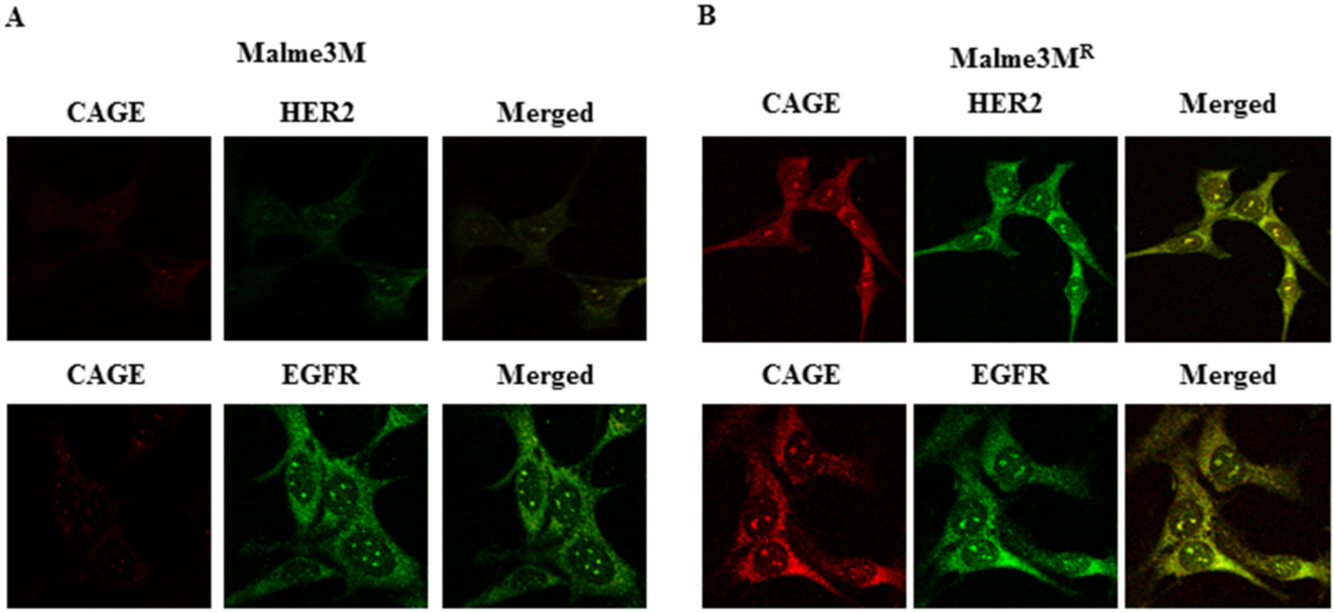

Supplementary Figure S3: CAGE shows co-localization with EGFR and HER2 in Malme3M^R cells. Immunofluorescence staining employing the indicated antibody was performed in Malme3M cells (**A**) and Malme3M^R cells (**B**).

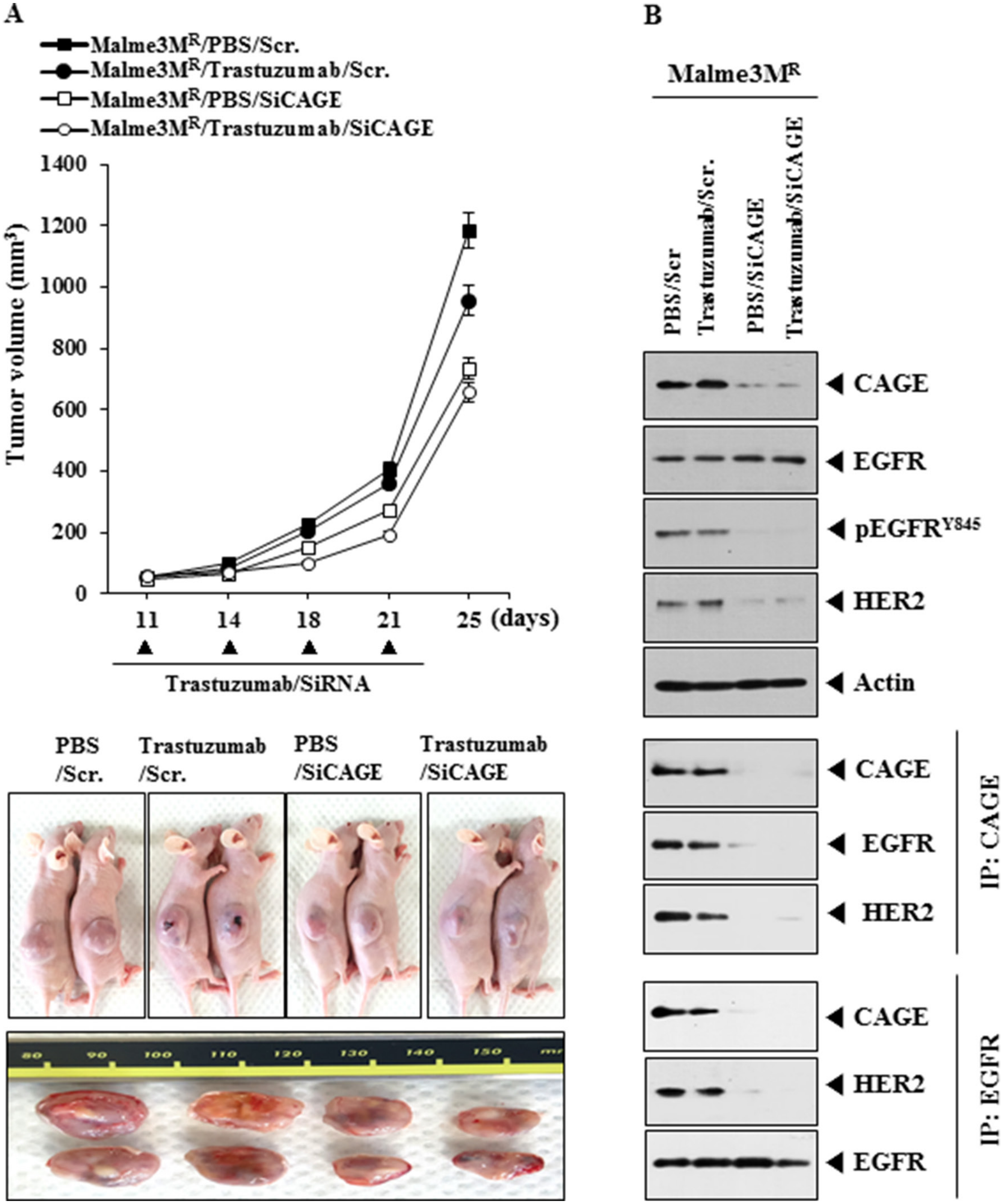

Supplementary Figure S4: The down-regulation of CAGE decreases the tumorigenic potential of Malme3M^{R} cells and enhances *in vivo* sensitivity to trastuzumab. (**A**) Malme3M^{R} cells (1×10^6) were injected subcutaneously into the dorsal flank area of the mice. Following the establishment of sizeable tumor, trastuzumab (10 mg/kg) was administered via tail vein. To determine the effect of CAGE on the *in vivo* tumorigenic potential, scrambled siRNA (100 nM) or siCAGE (100 nM) was injected along with or without trastuzumab (10 mg/kg) via tail vein 4 times in a total of 25 days. (**B**) Tumor tissue lysates were subjected to immunoprecipitation and immunoblot analysis.

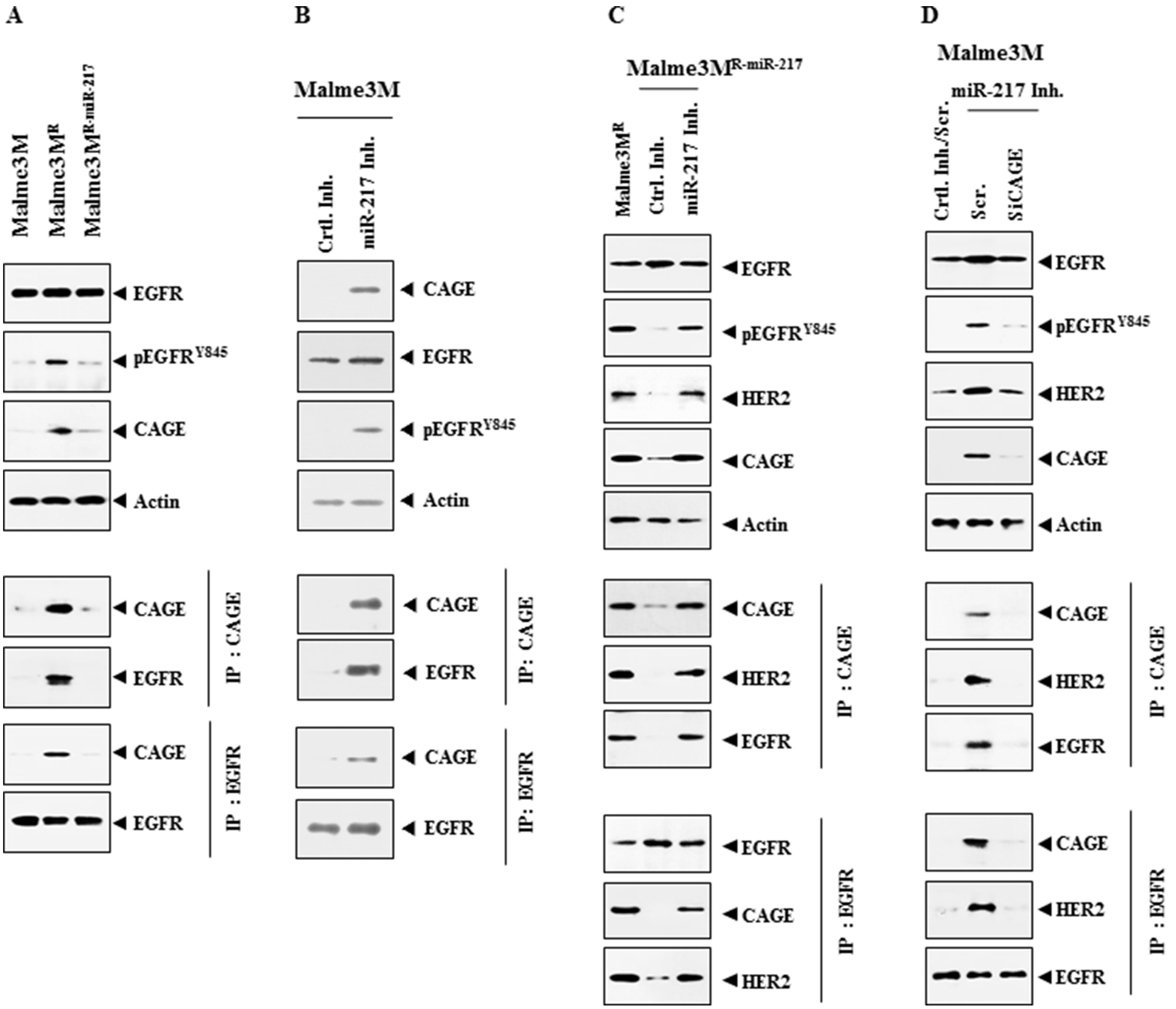

Supplementary Figure S5: miR-217 inhibitor increases the expression of pEGFR^{Y845} and induces the interaction between CAGE and EGFR. (**A**) Cell lysates prepared from the indicated cancer cell line were immunoprecipitated with the indicated antibody (2 μg/ml), followed by immunoblot analysis. Cell lysates were also subjected to immunoblot analysis. (**B**) Malme3M cells were transiently transfected with the indicated inhibitor (10 nM). At 48 h after transfection, cell lysates were immunoprecipitated with the indicated antibody (2 μg/ml), followed by immunoblot analysis. Cell lysates were also subjected to immunoblot analysis. (**C**) Malme3M$^{R\text{-}miR\text{-}217}$ cells were transiently transfected with the indicated inhibitor (10 nM). At 48 h after transfection, cell lysates were immunoprecipitated with the indicated antibody (2 μg/ml), followed by immunoblot analysis. Cell lysates form Malme3M^{R} cells were immunoprecipitated with the indicated antibody (2 μg/ml), followed by immunoblot analysis. Cell lysates were also subjected to immunoblot analysis. (**D**) Malme3M cells were transfected with the indicated inhibitor (10 nM) along with the indicated siRNA (10 nM). At 48 h after transfection, cell lysates were immunoprecipitated with the indicated antibody (2 μg/ml), followed by immunoblot analysis. Cell lysates were also subjected to immunoblot analysis.
